# Carbon-based iontronics – current state and future perspectives

**DOI:** 10.1039/d4sc06817c

**Published:** 2025-03-10

**Authors:** Panlong Li, Przemyslaw Galek, Julia Grothe, Stefan Kaskel

**Affiliations:** a Inorganic Chemistry I, Technische Universität Dresden Bergstrasse 66 01069 Dresden Germany stefan.kaskel@tu-dresden.de; b Fraunhofer IWS Winterbergstrasse 28 01277 Dresden Germany

## Abstract

Over the past few decades, carbon materials, including fullerenes, carbon nanotubes, graphene, and porous carbons, have achieved tremendous success in the fields of energy, environment, medicine, and beyond, through their development and application. Due to their unique physical and chemical characteristics for enabling simultaneous interaction with ions and transport of electrons, carbon materials have been attracting increasing attention in the emerging field of iontronics in recent years. In this review, we first summarize the recent progress and achievements of carbon-based iontronics (ionic sensors, ionic transistors, ionic diodes, ionic pumps, and ionic actuators) for multiple bioinspired applications ranging from information sensing, processing, and actuation, to simple and basic artificial intelligent reflex arc units for the construction of smart and autonomous iontronics. Additionally, the promising potential of carbon materials for smart iontronics is highlighted and prospects are provided in this review, which provide new insights for the further development of nanostructured carbon materials and carbon-based smart iontronics.

## Abstract 2

Carbon-based iontronics is an emerging field. Various nanostructured carbon materials enable bio-inspired information sensing, processing, and actuation by mimicking bioelectric signals and bioactive ion interactions.

## Graphical abstract

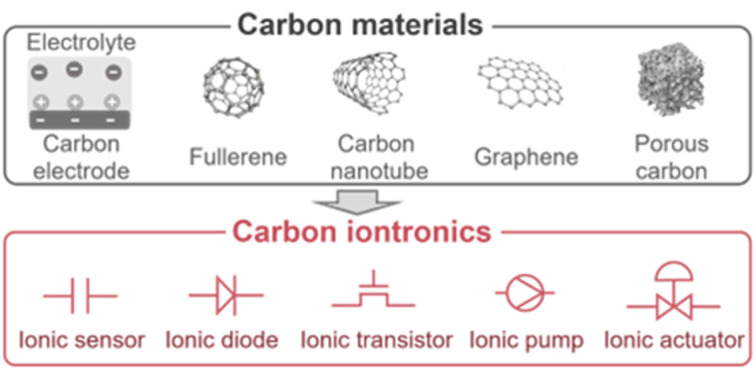

## 1. Introduction

The concept of iontronics was originally promoted by Leger, Berggren, and Carter in the book “Iontronics: Ionic Carriers in Organic Electronic Materials and Devices” in 2010.^1^ A strong motivation is based on mimicking nature in which neural systems mainly operate through ions instead of electrons. Despite being intrinsically limited in speed, in particular, the huge complexity of chemical ions, neurotransmitters, hormones, *etc.* regulated through electrochemical potentials over membranes and charge transport processes enables signal processing surpassing that of modern computing architectures by orders of magnitudes in terms of energy efficiency.^2^ Reducing the CO_2_ footprint of today's computing and digitalization mania remains among the most pressing challenges of the century.^3,4^

The original definition of iontronics mainly focused on organic semiconductor materials with ionic carriers. From this time the field of iontronics has greatly extended and is not restricted to organic and semiconductor materials but now refers to the essential and fundamental definition of the ion-electron coupling process in materials and devices. The distinction between iontronics and electronics relies on the charge carriers; electronics use electrons and holes, but iontronics operate through ions. For example, some electronic devices were constructed using innovative conductive materials (such as carbon materials) to realize similar iontronic functions (such as sensors,^5^ transistors,^6,7^ diodes,^8^ and actuators^9^), but their charge carriers were still electrons and holes, instead of ions.^10^ Although these electronic devices exhibit similar characteristics and functions to iontronics, they cannot be classified and defined as iontronics and will not be discussed in this review in detail.^5,11^

**Fig. 1:**
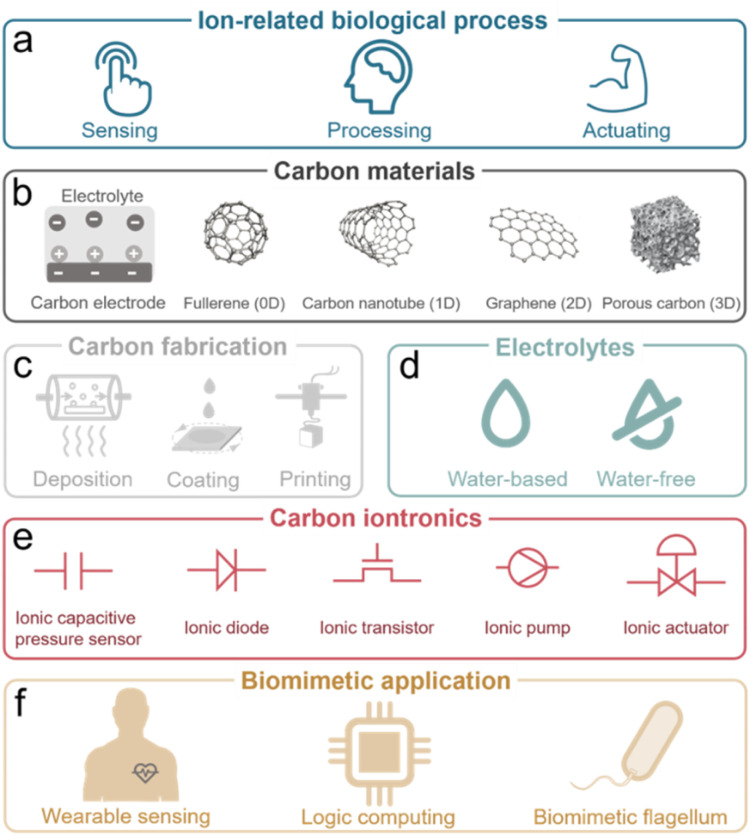
Carbon-based iontronics from (a) biological processes, (b) carbon materials, (c) fabrication, (d) electrolytes, (e) iontronic elements to (f) biomimetic applications.

Through ion-electron coupling processes (charge transfer, storage, or interaction), ion behaviors can be revised and regulated using a modulated electric signal.^10,12^ Ion movement can also generate an electric signal. This signal can be further detected and processed. These ion-electron coupling processes are inspired by the naturally evolved ion-related biological processes, ranging from sensing, processing, and actuating as illustrated in Fig. 1a and Section 2.^13–15^

Established iontronic devices face a series of material-related challenges, such as the balance of high electronic conductivity and ion transport efficiency, long-term chemical and mechanical stability in solution environments, sustainability, easy processability and miniaturization. Carbon materials, ranging from fullerenes (0D), carbon nanotubes (1D), graphene (2D), to porous carbons (3D), have seen tremendous development and great success in industrial applications due to their unique physical and chemical characteristics.^16–19^ The electronic and chemical bonding structures (highly ordered lattice structure) endow carbon materials with efficient electron transport channels,^18^ along with chemical, electrochemical, mechanical, and thermal stability. Nanostructured carbons (*e.g.*, graphene and CNTs) offer high surface area and tunable pores, improving ion mobility and selectivity. Carbon materials have proven to be excellent candidates for deliberately manipulating ions including in electrochemically stimulated electroadsorption, transport, faradaic processes, *etc.* through electric signals in the iontronic devices (Fig. 1b).^20–23^ The physical and chemical properties of carbon materials will be discussed in detail in Section 3.

In addition, carbon materials, derived from abundant and renewable resources (sustainability), can be further processed into various forms (thin films, powders, and composites) with nanometer-scale features (miniaturization) through a variety of technical methods (easy processability), including deposition, coating, and printing methods, have been developed for device construction, which allow for the integrated, miniaturized, and flexible carbon-based iontronics (Fig. 1c).^24–26^ Advanced techniques of carbon material processing will be further summarized in Section 4. As the core part of iontronics, various types of electrolytes are crucial for the development of iontronics (Fig. 1d). Therefore, we will summarize the development in the application of different ions and electrolytes in Section 5, including inorganic ions/bioactive ions in aqueous electrolytes, non-aqueous electrolytes, ionic liquids, gel electrolytes, and solid electrolytes.

We focus on five representative elementary carbon-based iontronic elements which are introduced and classified as ionic capacitive pressure sensors,^27^ ionic diodes,^28^ ionic transistors,^29^ ionic pumps,^30^ and ionic actuators^31^ (Fig. 1e), as described in Section 6. Hereafter, we will introduce bioinspired iontronic devices for sensing,^32^ processing,^33^ and actuating^34^ functions and promising integrated intelligent iontronic devices, such as the artificial afferent nerve (Fig. 1f and Section 7).

## 2. Ion-related biological processes and ion controlling strategies

**Fig. 2:**
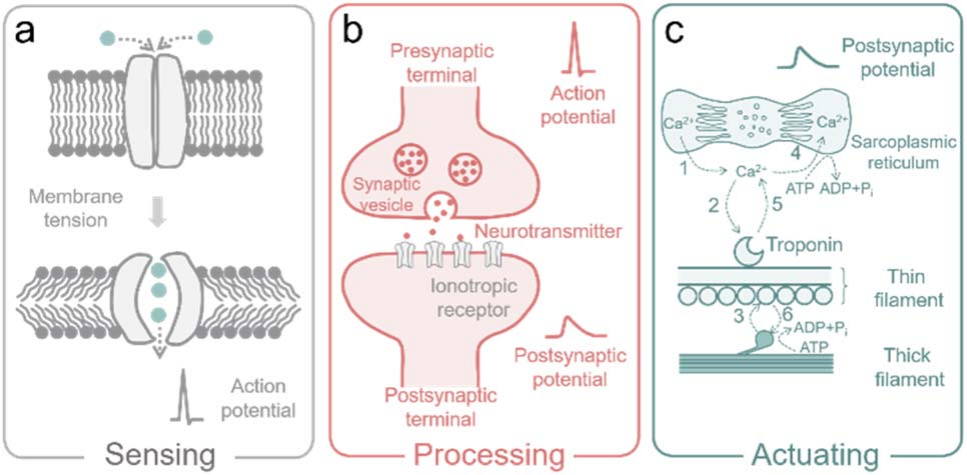
(a) The mechanosensitive ion channels in sensory neurons. (b) The synaptic transmission between presynaptic and postsynaptic terminals. (c) Ion-involved muscle shortening and lengthening.

In nature, living organisms receive and respond to environmental signals *via* various ion-involved biological processes, ranging from information sensing, processing, to actuating (Fig. 2), which are connected by the generation, propagation, and modulation of action potentials in the neuro systems. In particular, information sensations and perceptions such as mechanosensation rely on the mechanosensitive ion channels in the sensory neurons to convert physical stimuli (ranging from skin contact and sonic waves to gravity) into electric signals.

As shown in Fig. 2a, membrane tension (induced by mechanical force) leads to the structural switch of the mechanogating channel, causing the closure/opening of the ion path. Then the flow of ions through the opened ion path produces an electric signal (*i.e.*, active potential).^15,35^ The active potential will be propagated along the axon of the neuron with activated sodium channels as the motor. When the active potential arrives at the presynaptic terminal, the vesicles (with neurotransmitters inside) at the presynaptic terminal are forced to migrate, fuse with the outer membrane, and release the neurotransmitter (Fig. 2b). These neurotransmitters interact with the specific ionotropic receptor in the postsynaptic terminal, and then cause changes in the membrane potential of postsynaptic cells (so-called postsynaptic potential (PSP)), as shown in Fig. 2b.^13^

After processing of information in the neuro system, motor neurons release acetylcholine (ACh), which generates an active potential and leads to the release of Ca^2+^ from the sarcoplasmic reticulum (specialized endoplasmic reticulum in muscle cells for regulating Ca^2+^). After that, Ca^2+^ ions bind to troponin (a protein complex in muscles that helps regulate contraction) and activate the actin-binding sites in the thin filament of the muscle cell (actin is the globular protein that forms thin filaments).^36^ Then, myosin (the motor protein that forms thick filaments) pulls the actin chain and generates a force driven by triphosphate (ATP).^36^ When active potential stops, Ca^2+^ ions are then pumped back into the sarcoplasmic reticulum, leading to the detachment of the cross-bridge (a temporary bond between myosin and actin during muscle contraction) (Fig. 2c).^14^ Metal ions and neurotransmitters play crucial roles in the above-described ion-involved biological processes, which occur in mammal bodies every day. The fundamental mechanisms of these ion-relative biological processes inspire the development of ion-controlled devices (also termed iontronics) to mimic the biological functions and build advanced iontronics, including ionic capacitive pressure sensors, ionic diodes, ionic transistors, ionic pumps, and ionic actuators, for multiple biological applications. In the following sections, we summarize and review the ion behavior in the carbon-based bioinspired iontronics.

**Fig. 3:**
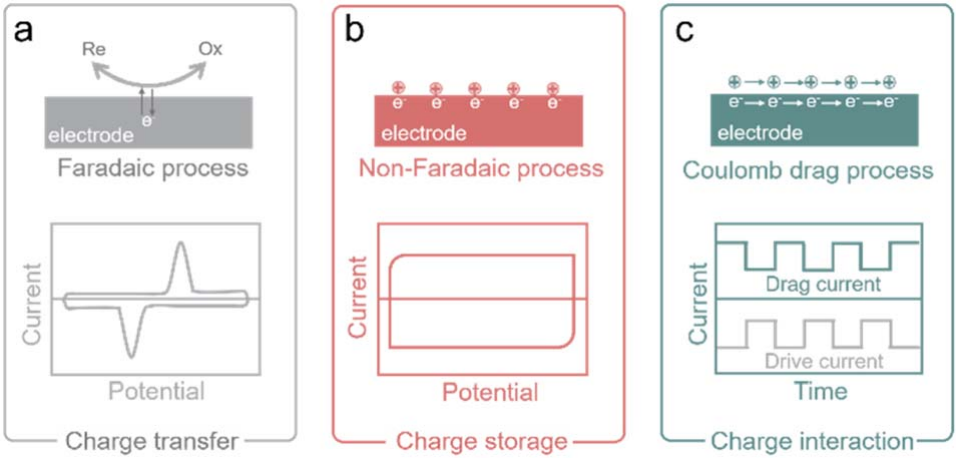
Ion-electron coupling processes in carbon-based iontronics. (a) The illustration of charge transfer in the faradaic process (top) and the typical CV curve of this process (bottom). (b) The illustration of charge storage in the non-faradaic electroadsorption process (top) and the typical CV curve of this process (bottom). (c) The illustration of charge interaction in the Coulomb drag process and drive current of electron flow in the electrode (top) and the relative drag current of ion flow in the electrolyte (bottom).

The ion-electron coupling enables the ion control and manipulation of carbon-based iontronics, which features multiple functions of iontronics, differing from conventional electronics without ion participation but electrons and holes. We have summarized three important ion-electron coupling processes, charge transfer, charge storage, and charge interaction processes (Fig. 3).

The charge transfer process is also termed a faradaic process, which involves the transfer of electrons between the conductive bulk phase of an electrode and the ions in an electrolyte at the interface of the electrode and the electrolyte (Fig. 3a). The electron transfer causes redox reactions, and such reactions obey the Faraday law, that the mass (*m*) of consumed or produced chemicals on the electrode is proportional to the charge (*Q*) through the electrode.^37,38^ The metal plating/stripping process is the typical process of charge transfer, which is dominated primarily by the redox reaction at the interface of the electrolyte and the conductive electrode.^39^

The charge storage process is also called a non-faradaic process, or electroadsorption, in which no electrons are transferred through the electrode and electrolyte interface, and no redox reaction occurs (Fig. 3b).^37,38^ Under the applied voltage, cations and anions move toward the surfaces of the polarized electrodes with opposite polarization respectively, and charges accumulate at the electrode/electrolyte interfaces to form an electric double layer (EDL). The EDL can be considered as a parallel plate capacitor, assuming that the counter charge of ions forms the second plate of the capacitor, and the charge is opposite to that of the first plate.^40^ A carbon-based supercapacitor (SC) exhibits typical charge storage characteristics, which store charges at the electrode/electrolyte interfaces. However, charge storage and charge transfer processes are also concomitant with the actual electrochemical process. For example, the EDL effect can also be found in the metal plating/stripping process, while redox reactions widely occur in carbon-based SCs due to the functional groups in the carbon surface (also termed pseudocapacitors).^37,41^

Besides two normal electrochemical processes, we additionally introduce a third ion-electron coupling strategy, which is called the Coulomb drag process (Fig. 3c). Coulomb drag refers to the long-range Coulomb interaction of charge carriers in two conductors that are close to each other but electrically isolated. When the electric current passes across one conductor (drive current), a current in a closed circuit (or a voltage in an open circuit) is thereby produced in the other conductor (drag current). The mobile charges in the “drive layer” power the quiescent charge in the “drag layer” and ion transport can be motivated near the conductive carbon surface when the current flows through the “drive layer” of carbon, and the ion transport can also drive the relative drag current.^12,42^

## 3. Carbon materials

Carbon materials, ranging from fullerene (0D), carbon nanotube (1D), graphene (2D), to porous carbon (3D) have unique physical and chemical characteristics, which predispose them to be excellent candidates for manipulating ion and electron behaviors in iontronic devices.^20,43–45^ We summarize the properties of these carbon materials in Table 1.^46–50^ They owe these specific properties to their structure, which is discussed for each material in this section. Recent advances in nanoscience have greatly increased prospects for the use of carbon-based nanomaterials in all aspects of human life.^51^

**Table 1:** Properties of fullerenes, CNTs, graphene, and porous carbons^46–50^

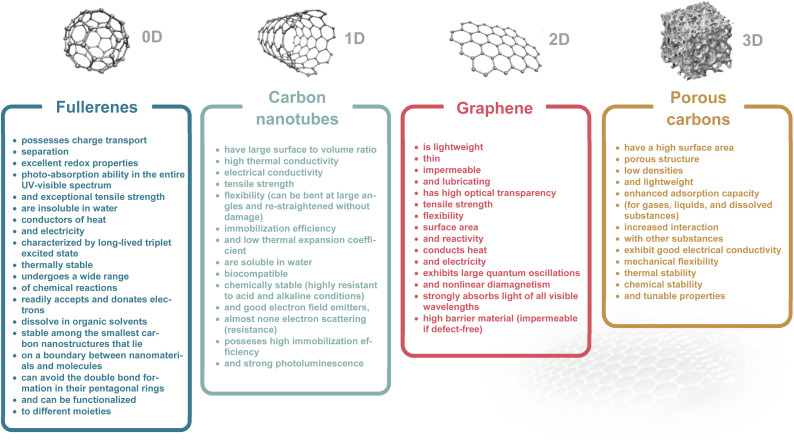

### 3.1. Fullerenes

Fullerenes (called “buckyball” structures) belong to zero-dimensional (0D) nanostructures where all dimensions are on the nanometer scale. They consist of carbon atoms connected by single and double bonds and form a closed or partially closed mesh.^52^ Fullerenes are not charged (or weakly charged), and have no unpaired electrons because of the absence of boundaries.^53^ High electron affinity of the molecule results in increased chemical reactivity towards the chemical addition reaction compared to planar graphene.^54^ The high electron affinity of fullerenes, when compared to that of oxygen molecules, highlights their strong electron acceptor nature, which is essential for iontronic devices that require the conversion of ionic signals into electronic signals. Their ability to be easily functionalized also allows for specific ion interactions, enhancing their utility in iontronic applications.^55^ Fullerenes have a long tradition in the development of organic solar cells and derivatives which are commercially available.^56–58^ Fullerene derivatives were applied as a novel humidity-sensitive material, inspired by the architecture of bacterial tentacles. This novel material is well suited for non-contact humidity monitoring because of its high hydrophilicity, quick responsiveness, and exceptional humidity sensing characteristics.^59^ Hydrophilic fullerene derivatives can serve as n-type active layers for organic electrochemical transistors (OECTs) with figures of merit exceeding those of the best-reported conjugated-polymer-based n-type OECTs. These thin-molecule films exhibit both facile ion transport and high electronic carrier mobility.^43^ Fullerenes and their derivatives due to their intrinsic electronic properties have a powerful capacity for radical scavenging.^60–62^ The ability to moderate the production of reactive oxygen species helps to prevent cell damage, such as mitochondrial injury and DNA fragmentation and makes them promising antioxidant agents for the treatment of oxidative stress-related disease.^63,64^ C_60_ fullerenes have also shown cytoprotective action against ultraviolet A (UVA) irradiation.^65^ Many recent studies have demonstrated the wide range of avenues of the use of fullerenes for their antioxidant, anti-viral, anticancer, and immunological properties.^66^ Furthermore, C_60_ fullerenes can be used as a nanocarrier for gene and drug delivery systems especially in cancer therapy.^67,68^

### 3.2. Carbon nanotubes

Carbon nanotubes (CNTs) belong to the one-dimensional allotropic group of carbon and can be considered as a rolled form of another allotrope of carbon called graphene. CNTs are hollow cylindrical structures with a hexagonal arrangement of carbon atoms that are sp^2^-hybridized. Their surface consists of single or multiple layers of graphene sheets; accordingly, they are called single-walled CNTs (SWCNTs) or multi-walled CNTs (MWCNTs). SWCNTs have caps at both ends, which can be removed to expose their hollow interior for usage as nanoporous materials, enhancing their suitability for ion transport or molecular storage applications. SWCNTs with an ultranarrow inner diameter (analogous to protein channels) are ideal candidate materials for building biomimetic ion pumps,^69,70^ and nanofluidic devices.^71,72^ SWCNTs are metallic or semiconducting depending on the chirality vector. Their bundled structures, which arise from aggregation, influence properties like ion accessibility and mechanical stability. Separation is expensive but possible through gel-chromatography^73^ or dielectrophoresis.^74^ Semiconducting SWCNTs are often used in electrolyte-gated transistors. They gain much attention in this type of transistor due to their regular porosity, which provides easy ion penetration and fast diffusion.^75^ Additionally, CNT sheets have been applied in electromechanical actuators for generating large stress and strain at several volts.^76^ Moreover, CNT composites have mimicked the function of artificial muscles^77^ and bionic soft robots in object motion.^78^ Besides, the elastomer/CNT composite demonstrates high deformation capability upon photothermal stimuli.^79^ In the field of nanomedicine, carbon nanotubes show significant promise as a nanomaterial for both therapeutic and diagnostic purposes. Various methods have been devised to make this substance biocompatible and to control any hazardous consequences that may follow. Chemically functionalized carbon nanotubes show less toxicity when used medicinally, but because of their apparent non-biodegradability, they are still viewed with skepticism.^80^ Biomolecules have been successfully integrated with CNTs, which enables the use of such hybrid systems as electrochemical biosensors (enzyme electrodes,^81^ immunosensors^82^ or DNA sensors^83^).

### 3.3. Graphene

Graphene is an isolated atomically thin layer of graphite with a 2D honeycomb (hexagonal) crystal lattice.^84,85^ Graphene consists of sp^2^-bonded C-atoms arranged in a plane with a molecular bond length of 0.142 nm. In graphene, the arrangement of carbon is trigonal planar where one carbon atom is coupled with the other three adjacent carbon atoms by σ-bonds and a delocalized π-bond, which contributes to a valence band that extends over the whole sheet (the valence band is touched by a conduction band).^86^ Graphene with a tiny overlap between valence and conductance bands and electron mobility as a 2D carbon material, has been widely explored in the field-effect transistor (FET) field over the last few decades.^87^ Graphene is known for its exceptional carrier mobility and the ability to be functionalized with various chemical groups. This makes graphene highly efficient in iontronic devices, where quick and responsive electronic transitions in response to ionic changes are required. Because of its well-established, straightforward production techniques, superior electrical and electrochemical properties, and availability, graphene and its derivatives are widely used as electrode materials for ion actuators.^88^ Moreover, because of its good chemical stability, graphene can be found in iontronics which operate in extremely acidic or alkaline environments.^89,90^ Additionally, graphene-based highly stretchable strain sensors have relatively stable conducting performance under various humidity conditions and sensitivity to strain variations.^91^ The unipolar ion transport within a 2D nanofluidic material and asymmetric charge distribution could be used to generate osmotic energy.^92,93^ Additionally, 2D graphene has been extensively explored as a platform for graphene-based FET biosensing and detection applications (such as antigen, protein, DNA, RNA, *etc.*), leveraging surface modification and ion-selective interactions.^94^

### 3.4. Porous carbons

Porous carbons are composed primarily of carbon atoms arranged in a three-dimensional (3D) network with a significant volume of pores and voids within their structure. They typically achieve specific surface areas up to 3000 m^2^ g^−1^ which surpasses that of all other carbons discussed above. The porosity of porous carbon can be classified into several categories based on the size of the pores. Microporous carbons have pores with width ≤2 nm. They are typically produced from organic precursors (wood, coconut shell, asphaltenes, *etc.*) through pyrolysis and chemical or physical activation.^95,96^ Carbon molecular sieves have tailored pore sizes with precise adjustment in the range of ±0.1 nm. An innovative approach to microporous carbons is silicon (metal) extraction from carbides by chlorination. However, the highly controllable pore diameter in carbide-derived carbons (CDCs) compensates for the high ecological footprint at least for fundamental research. An interesting feature is the availability of microporous carbons in various morphologies (powders, nanospheres, fibres, thin films, *etc.*). Mesoporous carbons have pores with width between 2 and 50 nm. Since the discovery of ordered mesoporous materials by Beck *et al.*^97^ the field has made huge progress today, and a wide variety of highly controlled ordered mesoporous carbons is accessible though soft- or hard-templating techniques providing excellent control of pore size, ordering, and even morphology.^98^

Macroporous carbons have pores with width ≥50 nm. They typically have a more open structure and higher permeability than microporous and mesoporous carbons. There also exist hierarchically porous (micro–meso–macroporous) carbons with ordered/disordered porosities and amorphous/graphitic textures.^49,99^ Porous carbons have been widely used in electrochemical capacitors (ECs) and are an essential component for industrial application.^100^ Particularly, the 3D porous structures with high specific surface area significantly increase the ability to store ions (mainly by EDL formation).^101^ The EDL effect plays also an important role in iontronic devices.^102,103^ Most porous carbons contain several pore-size fractions that provide facilitated ion transport. Meso- and macropores are important transport channels through which ions can move rapidly, while micropores are the target site for their electroadsorption and EDL formation.^104^

Porous carbon materials have gained significant attention for their biocompatibility in tissue engineering, drug delivery, and biosensing. The biocompatibility of porous carbon materials is largely influenced by their surface properties and pore structure. The surface can be modified to enhance cell attachment, proliferation, and differentiation, making them suitable for scaffolding in tissue engineering.^105^ Studies have shown that porous carbon materials exhibit low cytotoxicity, meaning they do not harm cells in contact with them.^106^ Additionally, their hemocompatibility, or compatibility with blood, is crucial for applications such as implantable devices. For example, carbon-based scaffolds have been shown to support the growth of various cell types, including bone^107^ and stem cells,^108^ without eliciting significant immune responses. In drug delivery, porous carbon materials are valued for their ability to adsorb and release therapeutic agents in a controlled manner. The porous structure allows for high drug loading capacity and targeted delivery, reducing side effects.^109^

## 4. Fabrication of iontronic devices

**Fig. 4:**
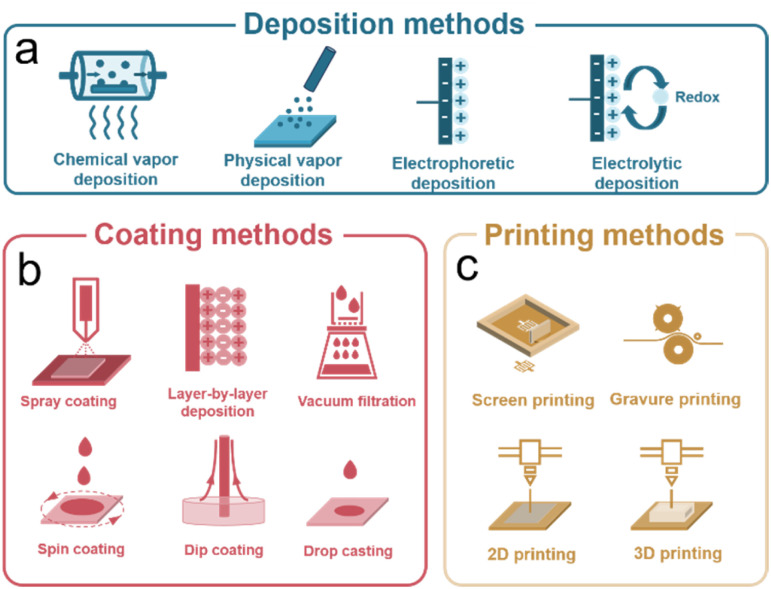
Fabrication methods of iontronic devices. (a) Deposition methods; (b) coating methods; (c) printing methods.

Carbon-based iontronics make use of carbon-based electrodes. The fabrication of the electrodes can be achieved through deposition, coating, and printing methods (Fig. 4). These methods offer flexibility in terms of material choice, device design, and scalability, allowing for the realization of a wide range of carbon-based iontronic devices.

### 4.1. Deposition methods

Deposition methods have proven flexible and powerful for fabrication of carbon-based devices, where the product self-assembles and deposits on a suitable substrate. Deposition techniques involve chemical vapor, physical vapor, electrophoretic, and electrolytic deposition (Fig. 4a).

Chemical vapor deposition (CVD) involves the growth of carbon-based materials on a substrate by decomposing gaseous precursor molecules. CVD is the most common for the fabrication of carbon-based iontronic devices due to its ability to produce high-quality thin films of carbon materials with controlled morphology and thickness.^110,111^

Furthermore, CVD can be used to deposit thin films of amorphous carbon^112,113^ or carbon nitride^114^ which serve as active components or coatings in iontronic devices. In addition, physical vapor deposition (PVD) can produce thin films by heating and vaporizing solid source materials to deposit them on the targeted substrate.^115,116^ PVD allows direct deposition onto targeted substrates *via* sublimating the graphitic sources which enhances top-down fabrication techniques to utilize samples in their as-grown state for device configurations.^117^ The important class of diamondoid thin films is not discussed here as their use in iontronics has been rarely explored so far.

Electrophoretic deposition methods have been documented for activated carbon,^25^ carbon nanotubes,^118^ graphene,^119,120^ graphene quantum dots,^121^ and onion-like carbon.^122^ They are cost effective, relatively fast, simple, and can be up-scaled using relatively simple fabrication equipment. Moreover, the deposited film thickness can be well controlled using this method. Electrophoretic deposition involves the diffusion, migration, and deposition of charged nanoparticles, nanotubes, or nanosheets onto pre-patterned current collectors by an electric field. Huang *et al.* proposed onion-like carbon based micro-supercapacitor (MSC) electrodes prepared by electrophoretic deposition combined with a eutectic mixture of ionic liquids (ILs), producing a MSC which is able to function from −50 to 80 °C.^122^

Electrolytic deposition uses redox reactions in a solution containing precursors, to induce growth of the electrode material under an electric field.^123–125^ The concentration of the electrodeposition solution and the deposition time need to be tightly controlled to avoid the growth of active materials in a lateral direction, which could lead to an electrical short circuit in iontronic arrays in series.^126^

### 4.2. Coating methods

Coating methods are crucial for the fabrication of carbon-based thin-film electrodes with high surface area, reproducible high structural homogeneity, and a tailored functionality. Common techniques include spray coating, layer-by-layer assembly, vacuum filtration, spin coating, dip coating, and drop casting (Fig. 4b).^26,127^ These methods involve depositing carbon dispersions onto substrates, followed by solvent evaporation to form solid films with controlled thickness and composition (made from organic, inorganic, or hybrid solutions), essential for iontronic applications.

Spray coating, widely used in fabricating carbon-based microelectrodes or thin films, utilizes a spray gun or airbrush to deposit carbon dispersions.^128–132^ The homogeneity and mass loading of active carbon materials depend on the substrate temperature, the distance between the substrate and the spray nozzle, and the deposition time. A key aspect in this method is that the droplets generated during spraying should not block the nozzle. An example of a device manufactured by spray coating is a foldable array of patterned graphene/ZnO nanoparticle UV sensors and asymmetric MSCs integrated on a paper substrate with patterned liquid metal interconnections.^128^

Layer-by-layer assembly enables the formation of multilayered carbon films and involves alternately depositing oppositely charged carbon nanoparticles (layers) onto a substrate, forming multilayered films with controlled thickness and composition. Moreover, this simple and cost-effective method can be used for large-scale deposition of active materials in films.^133–135^ An on-chip MSC pattern was obtained *via* layer-by-layer spray deposition of both manganese dioxide (MnO_2_) nanoparticle-coated carbon nanotubes (MnO_2_-CNTs) and MnO_2_ nanosheet-decorated reduced graphene oxide (MnO_2_-rGO) on mechanically robust, flexible polyethylene terephthalate.^133^

With vacuum filtration, a solid is separated from a liquid, resulting in a stacked dense carbon film, as electrodes with high energy density in iontronic systems.^136–140^ Several hybrid materials can be formed into uniform films using a range of homogeneous dispersions. Asymmetric interdigital microelectrodes with a planar geometry were directly manufactured through mask-assisted layer-by-layer filtration of electrochemically exfoliated graphene nanosheets for all-solid-state planar lithium-ion micro-capacitors.^138^

Spin coating involves the deposition of a carbon solution onto a substrate, which is then spun at high speeds to spread the solution evenly and form a thin film. Through a scalable, CMOS-compatible, high-wafer-yield spin-coating process, multielectrode modular high-energy-density MSCs containing reduced graphene oxide (rGO), GO-heptadecane-9-amine (GO-HD9A), rGO-octadecylamine (rGO-ODA), and rGO-heptadecane-9-amine (rGO-HD9A) were fabricated.^141^

Dip coating involves immersing a substrate into a carbon dispersion, allowing the solution to adhere to the substrate surface, and then withdrawing the substrate at a controlled rate to form a uniform film. This method is commonly used to deposit thin films of carbon-polymer composites, nanoparticles, or self-assembled monolayers onto substrates, enabling the fabrication of functional coatings or interfaces in iontronic devices.^142,143^ Su *et al.* used the dip coating method to prepare ultrafine Fe_3_O_4_ nanoparticles/graphene on carbon cloth for SC electrodes without any subsequent operations and extra additives.^142^

Drop casting involves placing small droplets of a carbon solution onto a substrate and allowing the solvent to evaporate, leaving behind a thin film or deposit. This method is particularly useful for depositing colloidal suspensions or functionalized nanoparticles onto substrates, enabling the fabrication of ion-selective membranes, sensing layers, or catalyst coatings in iontronic devices.^144,145^ Zhu *et al.* demonstrated a facile method for the fabrication of all-solid-state ion-selective sensors using drop casting. With block polymer F127 as the dispersant, multi-walled CNTs can be successfully dispersed in different polymer matrices and produce uniform sensing membranes.^144^

### 4.3. Printing methods

Printing methods are used to make interdigitated carbon-based microelectrode arrays by direct ink writing; these include screen, gravure, and two- and three-dimensional printing (Fig. 4c).^146,147^ These techniques save both time and materials, they are cost-effective, and they can be up-scaled.

Screen printing is a printing method where a carbon-based ink can be transferred onto a substrate using a mesh screen. During the procedure, a stencil is made on the screen to let ink flow through some places while obstructing it in others.^148,149^ Typical film thicknesses of carbon-based screen coatings range between 5 and 50 μm. This range can be adjusted based on the mesh count of the screen (a screen with a lower mesh count will deposit a thicker layer of ink; 100–200 threads per inch),^148^ ink viscosity (representing a fluid's resistance to flow), and composition.^149^ Hatala *et al.* examined the influence of the carbon-based ink composition on the functional parameters. The rheological properties of the wet samples along with the conducting properties, roughness, and mechanical resistance of the screen-printed dried layers were analysed.^149^

Another method is gravure printing, also known as intaglio printing to fabricate carbon-based coatings and functional films. Gravure is a high-quality printing process, where the structure to be printed is etched or engraved onto a substrate.^150,151^ The engraved areas hold the ink, while the non-engraved areas are wiped clean. Gravure printing is known for its ability to produce consistent and high-resolution structures. The properties of the ink, particularly its viscosity (0.01–1 Pa s), surface tension, and solid content, are crucial in determining the final film thickness (100 nm–10 μm) of a coating.^152^ The characteristics of the gravure printing process (cell geometry, squeegee pressure, and printing speed),^153^ the choice of substrate (ink spreading and adhesion), and post-printing processes (curing, sintering, or annealing) influence the final thickness of a film fabricated by this method. Gao *et al.* fabricated polypyrrole/graphene-based MSC arrays on printed paper through a facile and straightforward templating and imprinting strategy. By simply pressing the polypyrrole/graphene pieces into a mask, the MSC electrodes were easily embedded and fixed on the paper, which can effectively maintain the integrity and stability of electrode patterns.^150^

2D printing, also known as inkjet printing utilizes specialized printers to deposit (ejection) precise patterns of functional carbon-based inks or solutions from nozzles onto substrates. This method provides high-resolution patterns with precise control of the width, thickness, and distance.^21^ Inkjet printing can be used with versatile, environmentally friendly, and cost-effective precursors by creating small drops on various substrates. This method is a fast process at low cost. For an efficient inkjet printing process, the ink should have a high solid content and high stability. Moreover, printable ink formulations should have appropriate properties, such as the surface tension and viscosity of the ink. Inkjet printing is less advantageous for mass production but is ideal for rapid prototyping as any computer-generated pattern can be freely designed and realized. The film thickness in inkjet printing is generally much thinner (10–2 μm) than in other printing techniques like screen or gravure printing. The final film thickness is influenced by a combination of factors including the ink formulation (viscosity 1–40 mPa s, surface tension, and concentration of carbon materials), printer settings (droplet size, print resolution, and number of layers) substrate properties (surface energy, porosity, and ink temperature) and post-processing techniques (thermal annealing and sintering). With high-resolution inkjet printing, Gao *et al.* devised an iontronic touch sensing matrix with compliance, transparency, and strain insensitivity.^154^

Aerosol jet printing (AJP) is a very promising substitute inkjet printing additive manufacturing method that overcomes these two drawbacks.^155–158^ AJP is ideal for creating carbon-based interconnects, conductive films, and 3D microstructures, expanding its application toward advanced iontronic devices. The material to be deposited in AJP is suspended within tiny aerosol particles produced by the AJP ink and is subsequently guided in a patterned manner as a collimated stream onto the intended substrate. When an aerosol is used during the deposition process, the achievable viscosity range of AJP inks is expanded to 0.001–1 Pa s. This means that a greater variety of materials and larger solid content loadings can be deposited in comparison to inkjet printing. Furthermore, varying nozzle–substrate stand-off distances are made possible by the 3D gas flow wrapping that collimates the AJP aerosol particle stream, allowing for consistent deposition in a variable stand-off range of 1–5 mm. Because of this, AJP is ideally suited for deposition on tough substrates, even having the ability to print in three dimensions. Krzeminski *et al.* developed silver nanoparticle-based aerosol jet ink (AgNP ink), which is used to realize the construction of a hybrid circuit with aerosol jet printed pads and surface-mount devices.^155^

Iontronic devices, including customized electrodes and structural materials can be made inexpensively and versatilely *via* 3D printing.^159–162^ This method involves the extrusion of ink through a nozzle and is a type of additive manufacturing. The extruded material is deposited layer by layer, following a computer-generated design or model, to create a three-dimensional object. During the printing process, the extrusion nozzle moves along predefined paths, laying down the filament in a precise pattern to build up the desired shape. As each layer is deposited and solidified, subsequent layers are added on top until the object is complete. Extrusion printing is widely used due to its versatility and cost-effectiveness. It can produce parts with complex geometries and is suitable for prototyping, custom manufacturing, and low-volume production. High-viscosity inks with shear-thinning rheological characteristics are needed for this. These inks need to have good stability and high viscosity, and they need to be a homogeneous dispersion of active materials. The typical layer thickness in 3D printing with carbon-based materials ranges from 10–500 μm.^163^ Factors such as ink or filament composition (the viscosity of carbon material content) printer settings (nozzle size (0.2–0.6 mm)), printing speed, layer height settings (50–500 μm), and post-processing conditions (curing or thermal annealing) critically influence the final film thickness.

## 5. Electrolytes

Biological processes occur in the aqueous environment of living organisms.^164^ Consequently, water-based electrolytes are required for biocompatible iontronic applications, such as wearable healthcare devices,^165,166^ microfluid devices,^167^ and human-machine interfacing.^168,169^ In the initial stage of iontronics, device performance is the priority, rather than biocompatibility, ease of fabrication, or flexibility.^170–172^ Therefore, water-free electrolytes are also applied in some iontronics with low requirements for biocompatibility and biointerfacing application.

### 5.1. Water-based electrolytes

Research in aqueous electrolytes for energy storage devices has made great progress in the last few decades. The rational design of aqueous electrolytes is utilized for the purpose of better, safer, and cheaper energy storage devices.^173,174^ However, as the core component of iontronics, the demand for ions and electrolytes is quite distinct compared to that of aqueous electrolytes for energy storage devices.^20,175^ For example, H_2_SO_4_, Li_2_SO_4_, and KOH aqueous electrolytes are widely applied in aqueous supercapacitors, due to their high ionic conductivity.^40^ However, H_2_SO_4_ and KOH are not desired because of their toxicity and biological incompatibility. In contrast, Cl^−^, Na^+^, Ca^2+^, K^+^, and complex biologically active ions (such as neurotransmitter ions and phytohormone ions) that participate in biological processes, have been investigated in bioinspired biocompatible and wearable iontronics.^13,176,177^

However, water-based electrolytes encounter water leakage and evaporation,^178^ which demand complicated encapsulation and fabrication processes for iontronic devices.^179^ Hydrogel electrolytes contain a polymer (*e.g.*, polyvinyl alcohol (PVA), or polyethylene glycol (PEG)) network and an aqueous electrolyte.^180^ Ion movement inside the hydrogel network enables ion conductivity. The hydrogel structures have high water retention, which partly overcomes the liquid-leakage problem of aqueous electrolytes.^178^ Moreover, the flexibility and stretchability of hydrogel electrolytes meet the requirement of biointerfacing between iontronics and biology (such as iontronic devices in contact with soft skin, the heart, and the brain) due to the soft tissues and cells of biology.^181,182^ Functionality including transparency, biocompatibility, self-healing ability, and biodegradability can also be achieved in polymer-based ionic hydrogels by molecular chain design of the polymer, which have been applied for a variety of versatile soft ionic conductors.^179,183^

### 5.2. Water-free electrolytes

At a high working voltage, organic electrolytes and ionic liquids are widely applied in iontronics (transistors, ionic diodes, and ionic actuators).^23,184,185^ However, the priority of organic electrolyte design is quite distinct for the energy and iontronics field. For example, the volume change of the electrode induced by the ion size causes the deformation of the ionic actuator (discussed in Section 6.5), which relies on the size difference of anions and cations.^22^ Another example is the ion-sieving effect in two carbon materials with small and large pore sizes of the ionic diode, CAPode (explained in Section 6.2).^23,172^ The rational design of electrolyte ions for such iontronics is not preferentially targeting the electrochemical performance, such as higher ionic conductivity and lower viscosity, but rather targeting the specific purpose of iontronic functions. Besides, organic solvent-based gel electrolytes have also been explored for the easy fabrication and minimization of iontronic devices.^186^ Besides the liquid and liquid-containing gel electrolytes, solvent-free solid-state electrolyte systems (including dry polymer electrolytes and inorganic solid-state electrolytes) have recently been explored for the nanoscale fabrication of iontronic devices, which shows great potential for highly integrated iontronics.^179,187^ Unfortunately, dry polymer electrolytes with good flexibility have low ionic conductivity at room temperature.^188^ Inorganic solid-state electrolytes have higher ionic conductivity at room temperature but are normally rigid and inflexible, which limits their application in flexible devices.^189,190^

## 6. Basic elements of carbon-based iontronics

In the above sections, we summarized the concept of bioinspired iontronics, properties of carbon materials, fabrication of iontronics, and types of electrolytes. In this section, we highlight the basic elements of carbon-based iontronics, including ionic capacitive pressure sensors, ionic diodes, ionic transistors, ionic pumps, and ionic actuators. These carbon-based iontronic elements must meet the following conditions: they must be fabricated from or composed of carbon materials, modulate the behaviors of ions and electrodes and feature biomimetic functionality. Devices which do not meet all of these criteria, metal electrode/polymer-based or inorganic salt-based iontronics (such as ionic memristors),^191–193^ bioinspired electronics (such as piezoelectric and piezoresistive pressure sensors),^32,194^ and carbon-based devices (such as a carbon-based energy storage devices),^195,196^ are not discussed.

### 6.1. Ionic capacitive pressure sensors

**Fig. 5:**
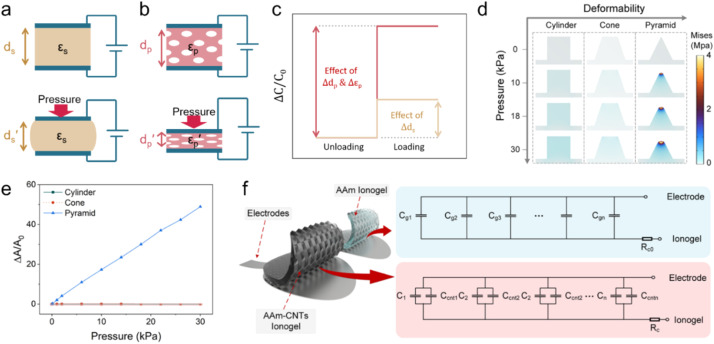
Schematic illustration of (a) a capacitive pressure sensor using a solid elastomeric dielectric layer, (b) a piezocapacitive pressure sensor using a porous elastomeric dielectric layer, and (c) capacitance changes in solid and porous elastomeric dielectric layer-based pressure sensors induced by identical loading. Reproduced with permission.^199^ Copyright 2016, American Chemical Society. (d) Results of finite element analysis of the stress distribution for different architectures (cylinder, cone, and pyramid) under varying pressures, (e) profiles of the contact area of microstructure electrode/electrolyte interfaces *vs.* applied pressures, and (f) the equivalent circuit of two ICPSs. Reproduced with permission.^27^ Copyright 2023, American Chemical Society.

Biology relies on mechanosensitive ion channels to sense mechanical force (such as airflow and sonic waves).^15,35^ The ionic capacitive pressure sensor (ICPS) has attracted a lot of attention for pressure-sensing technology,^197,198^ as shown in Fig. 5.

The sensor capacitance relies on the formation of an EDL at the interface of the electrode and electrolyte, which is determined by using eqn (1).^200^1
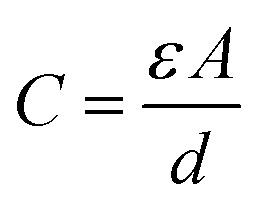
where *ε* is the dielectric constant (representing a material's capacity to store electrical energy) of the electrolyte, *A* is the overlap area of two electrodes, and *d* is the distance between two electrodes. Therefore, the mechanical force causes changes in these three factors of capacitive sensors, leading to detectable and sensible changes in capacitance.^200^

For the construction of better ionic capacitive pressure sensors (ICPSs), there are five main parameters for evaluation: high sensitivity, wide pressure range, low detection limit, short response time, and long life time.^200^

As a crucial factor, the sensitivity (*S*) is defined by using eqn (2),^27^2
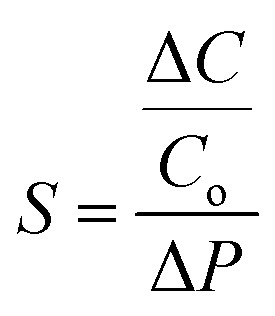
where *C*_0_ is the initial capacitance of the change in the sensor, and Δ*C* is the capacitance change when Δ*P* of external pressure is applied to the sensor. The enhancement of the sensitivity of an ICPS can be achieved by increasing the change in Δ*C* under applied pressure, which can be further derived based on eqn (1), as shown in eqn (3).3
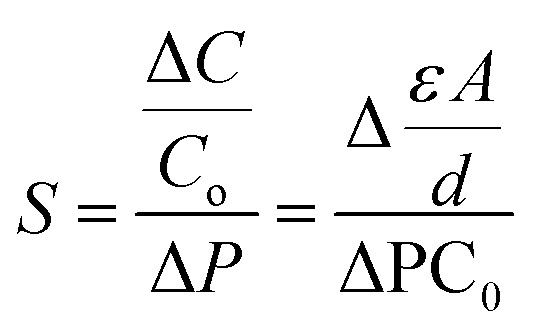


The change in Δ*C* can be adjusted by changing *ε*, *A*, and *d* according to eqn (3). For capacitive pressure sensors with a solid elastomeric dielectric layer (dielectric layer: electrically insulates different conducting layers or regions within a device) (Fig. 5a), the capacitance changes result from a decrease in the distance of the two electrodes (*d*_s_) under applied pressure while the dielectric constant (*ε*_s_) remains unchanged. To improve the capacitance changes under external pressures, modification of the elastomeric dielectric layer has been proposed *via* a 3D microporous dielectric elastomer (Fig. 5b).

Applied pressures compress the porous elastomeric dielectric layer (leading to a decrease in electrode distance (*d*_p_)) and increase the dielectric constant (*ε*_p_), which results in a significant capacitance increase compared to that of the solid dielectric layer (Fig. 5c).^199,201^

Compared with other transistor-based sensors and resistive sensors, ICPSs, consisting of two conductive electrodes with a dielectric layer inside, have simple structures and good compatibility for biological applications.^200^ The development of carbon-based ICPSs is focused on the modification of the electrode and dielectric layers. A polydimethylsiloxane (PDMS)/CNT flexible porous electrode with a 1-ethyl-3-methylimidazoline bis(trifluoromethylsulfonyl) imide ([EMIM][TFIS])-polyvinylidene fluoride-hexafluoropropylene copolymer (P(VDF-HFP)) electrolyte has been constructed to build a capacitive sensor and convert the pressure-induced deformation of the electrolyte/electrode interface into capacitance changes. In the initial state without external pressure, the porous hierarchical microstructure results in a small contact area of the electrode and electrolyte, and thus causes a small initial capacitance (*C*_0_). The applied pressure increases the contact area (*A*) and decreases the distance between the two electrodes (*d*), thereby greatly increasing the capacitance of the sensor. This well-designed porous hierarchical structure in the ICPS helps to achieve a high sensitivity of 131.21 kPa^−1^ with a wide pressure range of 0–1 kPa.^202^

However, the dimension and structure of microstructures (such as spherical and column arrays) still limit the good linear response and sensitivity because of their gradually saturated capacitance response. Consequently, Zhao *et al.* used finite element analysis to compare the deformation of three different surface architectures, cylinder, cone, and pyramid, under the same pressure conditions (Fig. 5d). The pyramid microstructure is found to have the most significant normalized area change (Δ*A*/*A*_*0*_) compared to the cone and cylinder microstructures (Fig. 5e). Compared with each pure acrylamide (AAm) gel (*C*_g_), there is an additional capacitance of *C*_cnt_, connected to *C*_g_ in parallel (Fig. 5f). Therefore, an excellent ICPS was built by combining CNT topological networks and ionic hydrogel micropyramid array microstructures that showed pronounced high sensitivity (1050–3775 kPa^−1^) within a wide pressure range (0–28 kPa).^27^

The EDL process normally has a limited capacitance, which limits the EDL-based ICPSs. Yoon introduced pseudocapacitive behavior based on the redox reaction of electrodes to enhance EDL capacitance by combining EDL-based CNTs and poly(3, 4-ethylenedioxythiophene):poly(styrene sulfonate) (PEDOT:PSS) with pseudocapacitance, which yields 2–3 times higher capacitance compared with EDL capacitance or pseudocapacitance alone.^203^ In addition, graphene, carbon black, and porous carbon have also been investigated in ionic capacitive pressure sensors,^201,204,205^ and the performance comparison of carbon-based ionic capacitive pressure sensors is summarized in Table 2.

**Table 2:** Summary and comparison of carbon-based ionic capacitive pressure sensors^a^

Electrode materials	Dielectric material	Sensitivity (kPa^−1^)	Response time (ms)	Pressure range (kPa)	Stability (cycle)	Reference
PDMS/CNT	[EMIM][TFIS]-P(VDF-HFP)	131.21	101	0–1	3000	202
Cu/CNT	NaCl-PEDOT: PSS-VSNP-PAAm hydrogel	301.5	197	0.3–63.3	5000	206
Carbon black-cellulose-glycerol	HEC-glycerol-NaCl-hydrogel	14750000	65	0–35	5000	205
Stainless-steel-CNT	LiCl-AAm hydrogel	1050	10	0.03–28	1000	27
Resilient bismuthine-graphene	H_2_SO_4_-PVA hydrogel	0.326	—	0.5–4.5	1000	201
PANI-nanoporous carbon- graphene	[EMIM][TFIS]-P(VDF-HFP)	9029	135	0–100	10 000	204

a VSNP: vinyl silica nanoparticle; PAAm: poly acrylamide; HEC: hydroxyethyl cellulose; Aam: acrylamide; PANI: polyaniline.

### 6.2. Ionic diodes

**Fig. 6:**
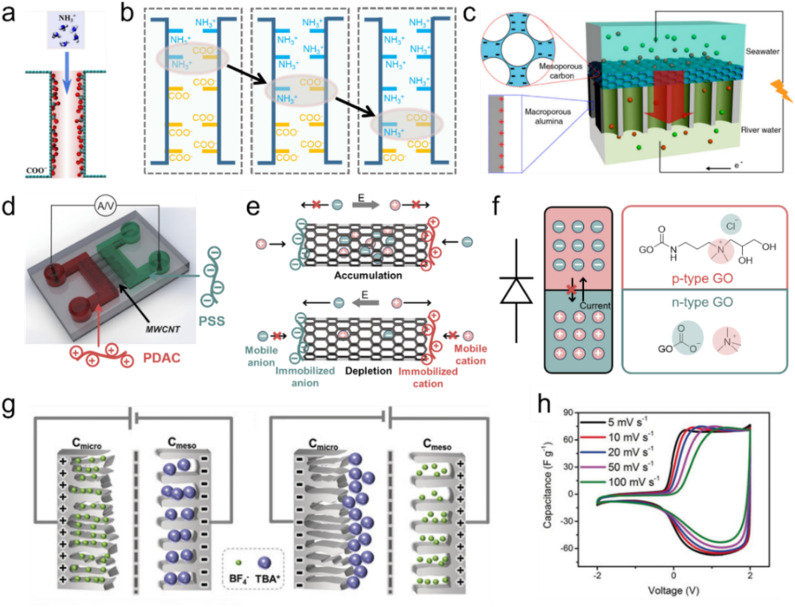
The mechanism summary of three types of carbon-based ionic diodes. (a and b) The molecular dynamics simulations of bipolar nanochannels. Reproduced with permission.^211^ Copyright 2023, American Chemical Society. (c) The bipolar ionic diode membrane. Reproduced with permission.^209^ Copyright 2014, American Chemical Society. (d and e) MWCNT-based bipolar ion channels (PDAC: poly(diallyldimethylammonium chloride); PSS: poly(sodium4-styrene-sulfonate)). Reproduced with permission.^212^ Copyright 2020, John Wiley and Sons. (f) Graphene-based p–n type ionic diode. (g and h) The capacitive ionic diode (also termed CAPode) is induced by ion-sieving effects and relative CV curves at different scan rates. Reproduced with permission.^172^ Copyright 2019, John Wiley and Sons.

A characteristic of an ionic diode is asymmetric conductivity, which is conductive in one direction and non-conductive in the opposite direction. The development of ionic diodes has recently been reviewed.^207,208^ However, carbon-based ionic diodes have rarely been discussed. In this part, we summarized three types of ionic diodes composed of carbon materials, including a bioinspired nanochannel diode,^209^ a p–n type diode (p–n type: a semiconductor structure with a combination of a p-type semiconductor (with an excess of holes) and an n-type semiconductor (with an excess of electrons)),^210^ and a capacitive ion-sieving diode^172^ (Fig. 6).

Functionalized biological ion channels, ranging from K^+^, Na^+^, and Ca^2+^ to Cl^−^ ion channels, regulate ion-selective transport through the cell membrane, which is endowed with physiological functions.^213^ For example, ion channels convert the salt concentration gradient to bioelectricity in the plasma membrane of electric eels.^214^ The controlled ion transport in biological ion channels has been widely mimicked *via* artificial nanochannels in recent years.^215^ The behavior of ion transport and ionic current rectification (ICR) in Janus graphene (a type of graphene with different functional groups on opposite sides) channels have been simulated using molecular dynamics simulations.^211^ The geometry of the graphene channel and surface charge have been considered for the evaluation of ICR. It is found that the ionic current undergoes switching on/off states with the change in the electric field; ionic current is switched on for opposite electric field directions. For NH_4_^+^ ions, the highest ICR ratio is achieved when the heterojunction position is located in the middle of the channels (Fig. 6a and b).^211^

The charged inner surface attracts counterions and forms an EDL, and the distance of such EDL layers determines the ionic current and the ICR ratio. A too small layer distance causes the EDL to overlap and block the ionic flux, but a large layer distance weakens the electrostatic effect of the EDL for ion transport, leading to a low ICR ratio.^207,216^ Li *et al.* realized the rectification of ionic current using carbon black nanoparticles to construct an asymmetric nanochannel membrane; the asymmetric nanochannel structure caused different ion concentration polarizations on different sides of the nanochannel.^217^ Gao *et al.* proposed the combination of mesoporous carbon (with a negative surface charge) and macroporous alumina (with a positive surface charge) for the construction of an ionic diode membrane that shows a very high ICR ratio of 450 (Fig. 6c).^209^ Inspired by the protein ion channels, Peng *et al.* developed a bipolar ion channel based on the decoration of oppositely charged polyelectrolytes on the MWCNT channel entrances (or the two CNT terminals) and achieved very high ICR (up to 1482) (Fig. 6d and e).^28,212^

Conventional electronic diodes rely on the p–n junction to realize the asymmetric conductivity for electrons (high conductivity in one direction for high current and low conductivity in the opposite direction for low current).^218^ Feng *et al.* first constructed an all-carbon material p–n junction diode with positively and negatively charged graphene oxides (GO), where p-type GO has tetramethylammonium cations (Me_4_N^+^) as mobile charge carriers and Cl^−^ anions are conducting ions in n-type GO.^210^ Under an external bias voltage, mobile Me_4_N^+^ cations (Cl^−^ anions) are transported from the p-type GO zone across the interface of p–n type GO to the n-type GO zone, leading to conducting current flux, while the current is blocked at the opposite bias voltage (Fig. 6f). Besides the GO-based p–n junction diode, Kaskel's group proposed a porous carbon-based capacitive ionic diode, which realizes a high ICR ratio due to ion size sieving in porous carbons with small and large pore sizes (Fig. 6g and h).^172^ The analogue of the diode semiconductor can be achieved in the capacitive device, the so-called CAPode (which stands for ‘‘capacitor’’ and ‘‘diode’’). Using different types of electrolytes with small cations (Na^+^/Li^+^) or small anions (BF_4_^−^), or polyanion-/polycation-based ILs, cation- or anion-dominated CAPodes have been constructed as P- or N-CAPodes, respectively.^23,219^ The mechanism and ICR of different types of carbon-based ionic diodes are summarized and compared in Table 3.

**Table 3:** Summary and comparison of carbon-based ionic diodes

Type of ionic diode	Mechanism for ionic rectification	The highest ionic rectification	Reference
Bipolar membrane	Heterojunctions of negatively charged mesoporous carbon and positively charged macroporous alumina	449	209
Asymmetric nanochannel membrane	Asymmetric nanochannel network membrane constructed from carbon black nanoparticles	110	217
Bipolar ion channels	MWCNTs decorated with oppositely charged polyelectrolytes at their two entrances	1482	28
p–n type ionic diode	Functionalized positively (p) and negatively (n) charged graphene oxides	6	210
CAPode	Cation and anion sieving in microporous and mesoporous carbon electrodes	12	172
CAPode	Polycation-/polyanion-based IL in an asymmetric carbon-loaded electrode	8	219

### 6.3. Ionic transistors

**Fig. 7:**
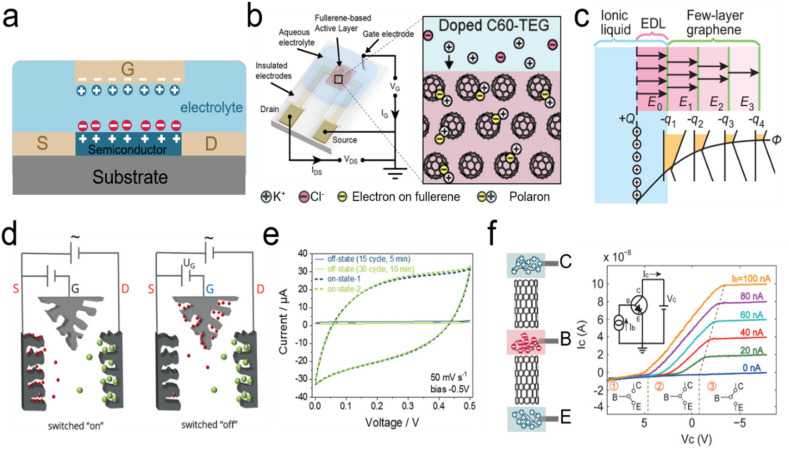
(a) The schematic diagrams of the electrolyte-gated ionic transistor (S: source, D: drain, and G: gate). (b) The ion-electron coupling mechanism of a fullerene derivative-based ionic transistor. Reproduced with permission.^43^ Copyright 2019, American Chemical Society. (c) Calculation of charge distribution for few-layer graphene under an electric field. Reproduced with permission.^220^ Copyright 2013, Springer Nature. (d-e) The schematic diagram of a switchable capacitor with gate characteristics (also termed G-Cap) and representative CV curves in the “on” and “off”-states. Reproduced with permission.^170^ Copyright 2020, John Wiley and Sons. (f) Ionic bipolar junction transistors fabricated *via* MWCNT-based bipolar ionic diodes and the corresponding *I*–*V* characteristics (C: Collector, B: Base, and E: Emitter). Reproduced with permission.^212^ Copyright 2020, John Wiley and Sons.

The conventional electronic transistor is a three-terminal element consisting of a drain (D), a source (S), and a gate (G). The electronic conductance (or resistance) of the semiconductor between S and D can be controlled by tuning the electric field *via* the gate terminal, which is separated by a dielectric/blocking layer. Therefore, controlling the external potential of G (*V*_G_) can modulate the current flow between D and S (*I*_DS_), allowing the transistor to be switched on/off (Fig. 7).^221^

Ionic transistors use ions as charge carriers, which is different from conventional electronic transistors which use electrons and holes. The ions in the electrolytes form an EDL at the interface of the electrolyte and electrode (G electrode/electrolyte and semiconductor/electrolyte) under an external voltage (*V*_G_). The EDL at the interface of the semiconductor and electrolyte causes the accumulation of electrons at the solid interface of the semiconductor, leading to changes in conductance (resistance) between the S and D electrodes and realization of transistor functions (Fig. 7a). Consequently, the ionic transistor is also called an EDL transistor, or electrolyte-gated transistor.^1^ And EDL formation in an electrolyte-gated ionic transistor (with SWCNTs as the semiconductor material) requires a much lower operating gate voltage (*V*_G_) and has a much higher channel conductance compared to the conventional solid-gated field-effect transistor.^222^

With regard to excellent physicochemical properties, carbon materials have been widely used as semiconductor channel materials with a tunable bandgap and high carrier mobility (such as SWCNTs, mono-graphene, and fullerene) or electrode materials (such as nanoporous carbon and carbon nanotubes) in ionic transistors. Fullerene derivatives have good electronic/ionic conductivity and have been proven to be excellent candidates for an n-type active layer for organic electrochemical transistors. Bischak *et al.* synthesized 2-(2,3,4-tris(methoxtriglycol)phenyl)[60]fulleropyrrolidine (C_60_-TEG) and glycolated side chains which facilitated the injection of ions into the fullerene-doped layer and greatly enhanced the conductivity of the semiconductor channel under external potential on the gate electrode (Fig. 7b).^43^ SWCNTs show both n-type and p-type characteristics for semiconductor materials, with ion gel as gate dielectrics. These transistors show an ultra-high switch on/off ratio of 10^4^, and a low gate voltage (lower than 2 V) with flexible features.^223^ Transconductances of SWCNT-based transistors exhibit temperature-dependent features, which endow them with thermal sensing applications.^224^ Furthermore, graphene appears as an excellent semiconductor material with high-density transport properties in electrolyte-gated ionic transistors, which has been reviewed in recent years.^1^ For graphene-based semiconductor materials in ionic transistors, the number of layers of graphene has influenced the conductivity of graphene in ionic transistors under the applied gate voltage. Ye *et al.* investigated the carrier density of mono-, bi-, and trilayer graphene in the ionic liquid-gated transistor, which showed the higher conductivity of the semiconductor channel with increasing graphene layers (from mono-to trilayer).^44^

For the EDL at the interface of electrolyte and graphene, the total capacitance (*C*) contributes to two capacitances in series, the geometric capacitance *C*_g_ (*C*_g_ = *ε*/*d*_EDL_; *ε*: the dielectric constant of the electrolyte and *d*_EDL_: EDL thickness) and quantum capacitance *C*_q_ (which comes from the shift of the Fermi energy of graphene by applying gate voltage), while 1/*C* = 1/*C*_g_ + 1/*C*_q_. Increasing the number of graphene layers leads to an increase in *C*_q_, but a decrease in *C*_g_. The optimal number of layers of graphene is 6 layers because multilayer graphene completely screened the applied electric field of the EDL effect (Fig. 7c).^220^ The physicochemical properties of graphene change with different ion concentrations and interactions which enables the multiple ion sensing functions of graphene-based ionic transistors.^225^

The EDL effect plays a crucial role in an ionic transistor. As the most widely used electrode materials for EDLCs, porous carbon materials are also showing promising applications in electrolyte-gated transistors, with porous carbon as a gate electrode, a much lower operating voltage and the high drain-source current modulation in the organic electrochemical transistor.^102,103^ Kaskel *et al.* proposed a novel all porous carbon ionic transistor (termed G-Cap) with ionic gel electrolyte; drain, source, and gate electrodes are made of porous carbon. The large gate electrode attracts ions from the channel of the source and drain (constituting the working capacitor) and leads to the decreasing capacitance of the working capacitor (Fig. 7d and e).^20,170,171^ Therefore, the capacitance of the working capacitor can be switched by controlling the gate electrode to build a switchable capacitor with transistor characteristics.^170^ Notably, 3D-printed in-plane G-Cap allows further integration with other printed iontronics.^20,171^

In addition to EDL-based ionic transistors, Peng *et al.* developed ionic bipolar junction transistors established *via* a MWCNT-based p–n-type ionic diode (Fig. 7f, left).^212^ The p–n configuration was achieved by PDAC or PSS polyelectrolytes. Two p–n junctions were connected to a three-terminal p–n–p-type transistor with a base (B) terminal modulating the currents between two terminals (C: Collector; E: Emitter). The “ON” and “OFF” status between C and E terminals can be controlled by the base terminal (Fig. 7f, right).^212^

### 6.4. Ionic pumps

**Fig. 8:**
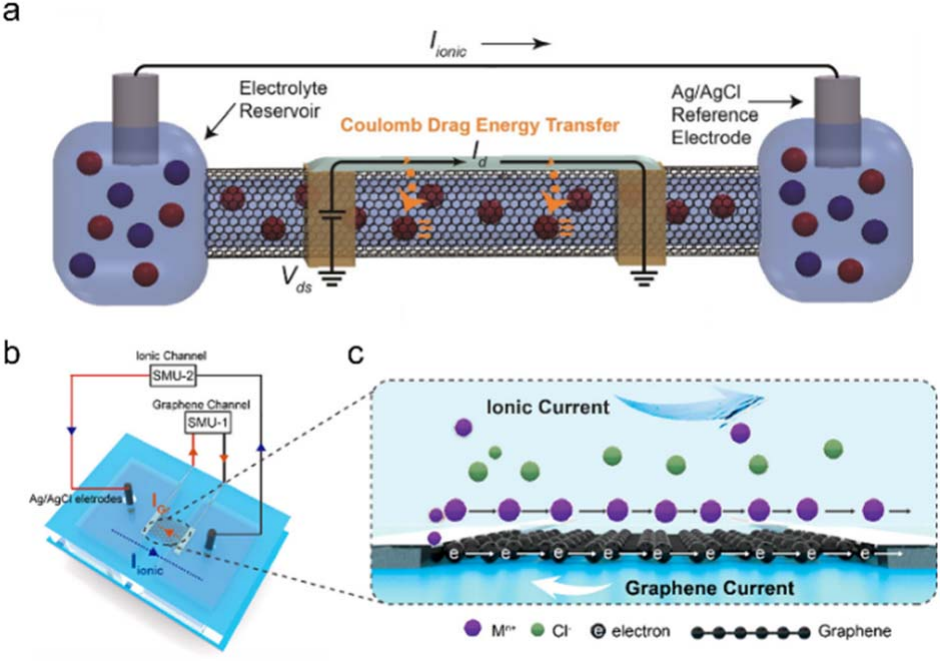
(a) The electronic current (*I*_d_) flowing along the SWCNT shell drives the ionic current (*I*_ionic_) flowing through the SWCNT core induced by the Coulomb drag process. Reproduced with permission.^30^ Copyright 2020, American Chemical Society. (b) The scheme of graphene-based ion-driven electrical current devices (current through the graphene (*I*_Gr_) and SourceMeter (SMU)) and (c) the scheme of an electric current in graphene driven by ionic flow. Reproduced with permission.^226^ Copyright 2023, American Chemical Society.

In biology, information signaling, transducing, transferring, and processing rely on accurate regulation of ions, especially incessant ion transport across cell membranes. Ion channels and ion pump structures in cell membranes play a central role in controlling ions into and out of cells (Fig. 8).^213,227^

The functions and features of ion channels and ion pumps are quite different. While ion channels control selected ions to diffuse rapidly down electrical and concentration gradients by one single gate opening/closing, ion pumps transport ions against gradients by consuming nucleoside ATP, which requires two collaborative gates and only one gate can open when the other is closed in the case of unrestricted ion flow.^227^

For bioinspired iontronics, ionic diodes and ionic pumps have been developed for different purposes. Ionic diodes are built for ionic current rectification that allows only for ion current to flow in one direction and blocks ion transport in the opposite direction; the ionic charge carrier can be cations, anions, or both.^207,208,210^ In contrast, ion pumps are used for ion transport and their delivery from the ion reservoir to the target zone and ionic current rectification is not the priority; the charge carriers for the ionic pump are the transported ions.^228,229^

Carbon-based ionic pumps, including SWCNTs and graphene, have been established for pumping ions.^71^ Specifically, the ultranarrow inner diameter of SWCNTs (analogous to protein channels) enables their widespread application in biomimetic ion pumps, artificial membranes, and nanofluidic devices.^230–233^ The mechanism of the ionic pump can be classified as electroosmotic and Coulomb drag processes. Electroosmotic-induced ion transport was achieved in the nanofluidic channels, where ion movement was modulated by an electric field through the channel of SWCNTs.^71^ Because of the negative charges at the CNT entrance (*i.e.*, carboxylic acid groups), the anion transport was blocked and only cations were selectively delivered under an external electric field. This SWCNT-based ion pump can not only pump small metallic ions (such as Na^+^, Li^+^, and K^+^) but also realize the transport of DNA macromolecules.^71,233^

Other ion-controlled transport has been explored based on the Coulomb drag effect. Coulomb drag refers to the long-range Coulomb interaction of charge carriers in two conductors that are close but electrically isolated. When the electric current passes across one conductor, a voltage (in an open circuit) or a current (in a closed circuit) is consequently generated in the other conductor. The mobile charges in the “drive layer” power the quiescent charge in the “drag layer” and ion transport can be motivated near the conductive carbon surface when the current flows through the carbon “drive layer”.^12,42^ Specific carbon-based ion transport devices have been established in the SWCNT ionic pumps by Rabinowitz *et al.* (Fig. 8a).^30^ When an external voltage was applied to the SWCNT shell, Coulomb drag coupling transduces electrical energy from the solid-state charge along the SWCNT shell to the electrolyte inside the SWCNT core. The electrical energy of the electric current flow along the SWCNT shell was transferred to the ionic current of electrolyte ions transported through the SWCNT core *via* the ion-electron coupling of Coulomb drag. The reverse process can also be observed in the graphene-based device (Fig. 8b and c).^226^ When the electric field produces ionic current near the electrolyte–graphene interface, the induced electric current can be detected across the graphene channel, and the electric current direction is opposite to the direction of the ionic current.

### 6.5. Ionic actuators

**Fig. 9:**
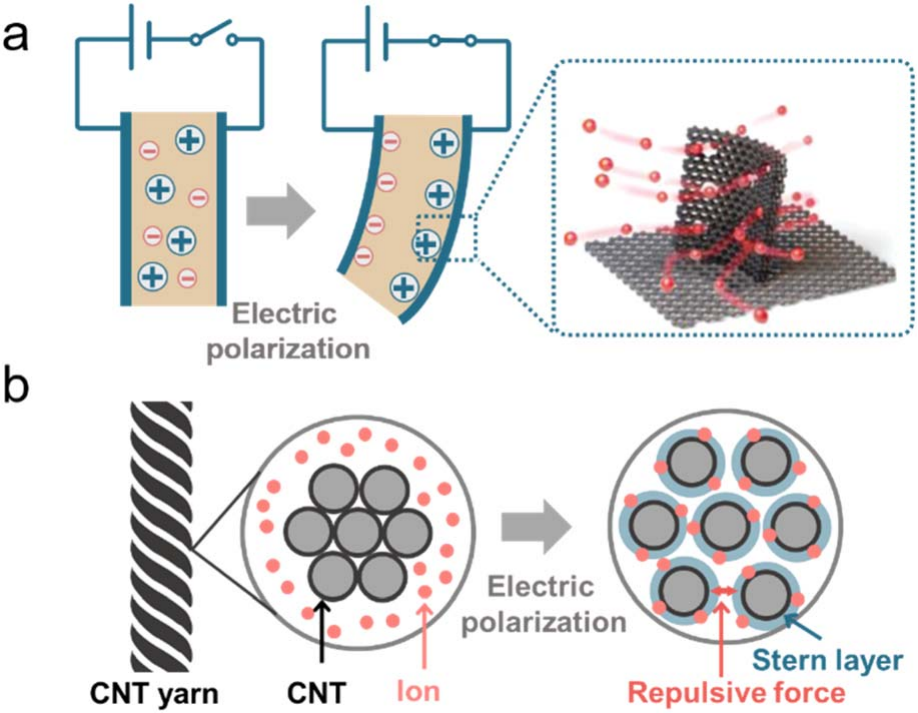
(a) The mechanism of bending actuators based on a 3D porous graphitic nanoribbon-graphene hierarchical porous structure. Reproduced with permission.^235^ Copyright 2020, John Wiley and Sons. (b) The mechanism of a CNT yarn-based ionic actuator. Reproduced with permission.^236^ Copyright 2024, Elsevier.

Biology of animals, microbes, and plants, relies on diverse integrated structures for ion motion and manipulation to realize ionic actuating functions and adapt to complex environments.^34,234^ Ion-motivated muscle shortening and lengthening lead to the deformation of the actuating structure of muscles and the hydrostatic skeleton, which is the basic actuating unit for manipulation and locomotion of animals.^34^ Carbon-based ionic actuators have been developed due to the excellent properties of carbon materials, as summarized in the previous section. In this part, we mainly introduce two types of ionic actuators, bending- and rotating-type actuators, which are both motivated by the volume deformation induced by the EDL effect (Fig. 9).^236,237^

Bending-type actuators are composed of two carbon electrodes (CNTs,^238^ graphene,^235^ and porous carbons^239^) and the electrolyte layer.^240^ For flexible and bending purposes, electrolyte layers normally contain solid polymer substrates and conducting ions of different sizes (such as EMIMBF_4_).^235^ The electric-induced shape change comes mainly from the dimension discrepancy of anions and cations. The positively polarized electrode repels large EMIM^+^ cations and absorbs small BF_4_^−^ anions, which leads to a volume compression of the electrode. The negatively charged electrode undergoes volume extension (Fig. 9a).^235^ In addition, the shape change of carbon materials can also result from the shrinkage of the average C–C bond length due to charge injection. It was demonstrated that the electron injection into CNTs or graphite caused electrode expansion and holes for slight contraction.^241,242^ Therefore, the deformation of the bending-type ionic actuator can be modulated by electric polarization. The bending deflection of the actuator is normally evaluated by using a laser rangefinder, which measures the horizontal distance from the initial vertical position to the bending position (under an external voltage), termed the peak-to-peak displacement.^22^ The displacement of the actuator can be controlled by modulating the values of applied voltages. The EDL plays a crucial role in bending-type ionic actuators, and therefore, modification of carbon electrodes with a high specific surface area along with fine electrical and electrochemical properties is desirable for better ionic actuators.^243^

In addition to bending actuators, rotating-type actuators have been developed as rotary motors that mimic the function of a flagellum (a tail-like part used for movement in some microorganisms). Such a rotating actuator is made of twist-spun carbon nanotube yarn (thinner than a human hair). When the CNT yarn was electrically polarized in the organic electrolytes, ions were injected into the yarn electrode and an EDL was formed on the CNT surface. The electrolyte ion injection and the repulsive force of the EDL lead to the volume expansion of the CNT yarn. The volume expansion of the yarn causes a decrease in its length, increasing diameter, and relative untwisting of the twisted structure (Fig. 9b).^236^ The simple bipolar CNT yarn can achieve a 15 000° rotation and 590 rpm in the organic electrolyte system reversibly.^45^ But bipolar CNT yarn cannot monotonically rotate over the available potential range, because anion or cation adsorption (during positive or negative polarization, respectively) of bipolar CNT yarn leads to similar volume expansion and untwisting. But unipolar stroke carbon nanotube yarn modified with cation exchange (or anion exchange) PSS or PDDA, respectively can shift the potential of zero charge in the opposite direction. Consequently, additive stroke changes can be obtained, and the stroke increases with increasing scan rates.^244^

## 7. Bioinspired and biomimetic applications

In Section 6, we illustrated five representative iontronic elements, and in this section, we introduce the promising bioinspired and biomimetic applications of these basic iontronic elements. For example, ionic capacitive pressure sensors,^200,245^ ionic diodes,^246,247^ and ionic transistors^248^ have been explored for sensing functions. Ionic diodes and ionic transistors are used for logic computing.^249,250^ In addition, ionic pumps and ionic actuators feature biomimetic actuating functions.^30,34,176^ In the following section, we summarize the bioinspired and biomimetic functions of carbon-based iontronics and introduce the integration of these elementary devices for further smart and autonomous iontronics (*i.e.*, reflex arcs).

### 7.1. Sensing

**Fig. 10:**
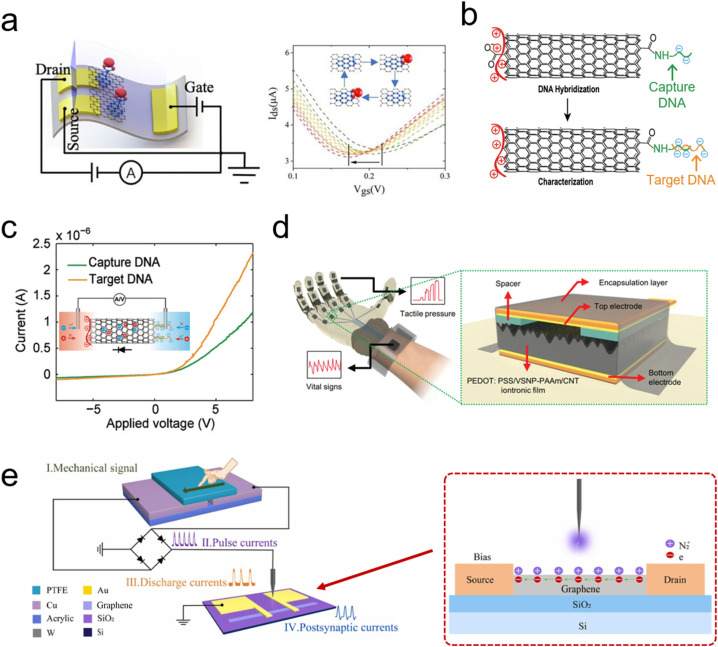
(a) Scheme of a flexible graphene-based ionic transistor for glucose detection (left) and the negative shift in the transfer characteristic curve of graphene in different concentration glucose solutions. Reproduced with permission.^251^ Copyright 2020, Elsevier. (b) Scheme of a MWCNT-based ionic diode for DNA detection and (c) *I*–*V* curves of a MWCNT-based ionic diode before/after target DNA hybridization. Reproduced with permission.^28^ Copyright 2021, John Wiley and Sons. (d) The scheme of a tactile perception glove with capacitive pressure sensor units. Reproduced with permission.^203^ Copyright 2022, John Wiley and Sons. (e) The scheme of a self-powered artificial mechanotactile sensing system, composed of a triboelectric nanogenerator and ionic-gated graphene transistor. Reproduced with permission.^252^ Copyright 2022, Elsevier.

In this part, we introduce two different sensing functions realized by carbon-based iontronics, including biosensing and pressure sensing (Fig. 10). Biosensing functions mainly rely on the graphene/CNT-based transistors to detect molecules/ions. The mechanism of a transistor-based biosensor is to detect the changes in the electric field on the gate electrode or channel caused by the ion interactions with the gate electrode of the channel (such as an electrochemical reaction); the changes in the electric field further affect the carrier density and current in the graphene- or CNT-based gate or channel, which can be detected in the transistor devices. Consequently, the concentrations of molecules/ions can be detected by measuring the changes in the channel current or gate voltage.^253,254^ Huang *et al.* constructed flexible graphene-based ionic transistors for glucose sensing. The graphene channel was modified by phenylboronic acid as the receptor, which enables the selective detection of glucose from human body sweat *via* wearable sensing iontronics. The negative shift in the transfer characteristic curve is a result of the binding of glucose to phenylboronic acid, which introduces n-type doping to graphene (Fig. 10a).^251^

In addition to widely explored ionic transistor-based biosensors,^255,256^ ionic diode-based biosensors have been also investigated, but carbon-based ionic diodes have been rarely reported.^208^ Recently, Peng *et al.* first proposed a carbon-based ionic diode for DNA detection, which was constructed by modifying with MWCNTs.^28^ MWCNTs with carboxylic groups on both terminals, were first modified with positively charged PDAC molecules (left terminal). The right terminal was functionalized with captured DNA molecules with negative charges (Fig. 10b).^28^ These captured DNA molecules were hybridized with the target DNA (with more negative charges than capture DNA), and the more negative charges result in higher ICR of such a MWCNT ionic diode (Fig. 10c). Consequently, biosensing functions have been realized in a CNT-based ionic diode.^28^

Besides biosensing functions, the sensibility and perceptibility of animal skin for the environment provide the necessary sensory interface between the living organism and changes in the external environment. Pressure, tactile, and force sensing are very important for skin sensory functions, such as physical touch, sonic waves, and liquid/airflow pressure.^32,257^ The development and evolution of biomimetic sensing techniques have greatly extended natural sensory functions (Fig. 10d and e). Flexible and wearable pressure-sensing devices have shown promising applications in the fields of tactile perception, wearable health care, and biomimetic robotics.^257–259^

Based on an ionic capacitive pressure sensor, Yoon *et al.* developed a series of pressure sensors for tactile detection, weak airflow sensing, and monitoring of vital signs.^203^ By fixing the sensing unit to the human hand, the tactile pressure of the hand can be detected by monitoring the capacitance changes in these iontronic sensing units (Fig. 10d). In addition, the high sensitivity of these iontronic sensor units enables the real-time monitoring of sphygmus and respiration of human. These ionic capacitive pressure sensors show good potential for wearable healthcare devices with their high sensitivity over a wide pressure range.

It should be noted that the electric signal obtained from these capacitive sensors is a capacitance change, which is a very low capacitance value (nF ∼ pF). This requires very sensitive and expensive instruments for signal registering and analysis. Not only iontronic sensors, but other electronic sensors also face similar problems, such as transistor-based sensors with ultralow current, and piezoresistive pressure sensors with ultralow resistance changes. Although high sensitivity can be obtained, the sensitivity requires advanced high-precision instruments, which hinders their further applications, such as integration, miniaturization, and economical efficiency.

Zhang *et al.* applied the triboelectric nanogenerator (TENG) to directly convert mechanical stimuli into electrical signals, thereby serving as a tactile sensor (Fig. 10e).^252^ The produced high-voltage electrical pulses further ionized N_2_ and produced N_2_^+^ ions, which formed an EDL in the graphene channel to modulate the conductance of the graphene channel of the transistor. In addition, the ion gel can be also used to form an EDL in these TENG devices.^260^ Overall, mechanical stimuli were converted into nonlinear artificial synapse signals in an integrated TENG and ionic transistor system without any external power supplier. However, the generated electrical signal information from the sensing process still needs a power source to be further processed and recorded for practical sensing applications.

Therefore, Peng *et al.* proposed a printed GO/rGO-based power storage device that produced energy by Li^+^ transport under a salinity gradient, and such a device can be charged by mechanical stimuli *via* a TENG.^261^ An all-in-one wearable health monitoring device was therefore built, composed of a power generation (TENG), an iontronic power storage system, and a wireless electronic sensing system.

Currently, integrated iontronic sensing devices are still in an early stage. Despite the complications and challenges associated with integrating iontronic sensors, the upsides of the attempt can be significant. The above studies provide a new concept for wearable and integrated information sensing and recording solutions. In the future, more efforts are needed to combine advanced iontronic sensing techniques (including pressure sensing, molecule sensing, temperature sensing, and humidity sensing) with power sources and information processing units to construct advanced integrated iontronic devices for e-skin,^262^ e-nose,^263^ health monitoring/diagnostics,^264^ and human-machine interfacing.^265^

### 7.2. Processing

**Fig. 11:**
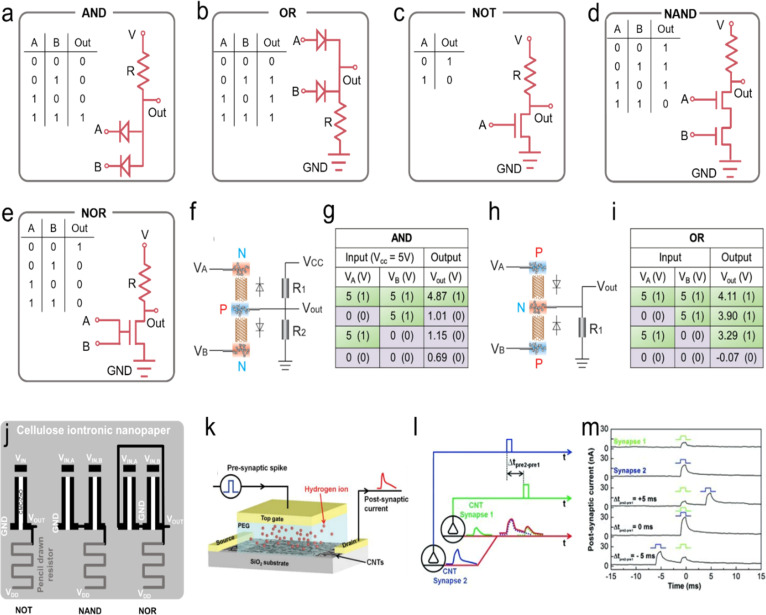
The circuit diagrams of logic gates and truth tables of (a) AND, (b) OR, (c) NOT, (d) NAND, and (e) NOR. Schematics of the (f and g) AND and (h and i) OR logic gates fabricated by using a CNT-based bipolar ion channel and the truth tables. Reproduced with permission.^212^ Copyright 2020, John Wiley and Sons. (j) Schematics of the logic gates (NOT, NAND, and NOR gates) fabricated by planar fully screen-printed carbon-based transistors with ZnO as the semiconductor channel and pencil-drawn graphitic load resistances as resistors. Reproduced with permission.^267^ Copyright 2022, John Wiley and Sons. (k) The structure of a CNT synapse (a pre-synaptic spike triggers a post-synaptic current, (l) schematics of two pre-synaptic spikes on two CNT synapses triggering EPSCs, and (m) current responses of the post-synapse (induced by different pre-synaptic spikes) *vs.* time. Reproduced with permission.^268^ Copyright 2012, John Wiley and Sons.

Information processing includes information computing, memorizing, and decision-making. The capability of information processing plays a crucial role in the adaptive survival of intelligent beings in complex and volatile environments. Animals rely on synaptic structures and naturally evolved neural networks in the neural system to process multiple and parallel external information (collected from the sensory system), which demands ultra-low energy consumption and simple synaptic structures due to the autonomous and decentralized computational mechanism, compared to conventional central processing units (CPUs).^187,266^ A simple logic unit, which can be integrated into iontronic devices, can work as a smart “brain” for intelligent functions to process the information obtained from the sensory part and convey the decision to the actuating components (Fig. 11).

Based on the basic elements of carbon-based ionic diodes and ionic transistors, simple logic circuits have been constructed for logic computation operations, ranging from AND, OR, NOT, NAND, to NOR (Fig. 11a–e).^267,269,270^ In a conventional electronic logic circuit, the combination of these basic logic circuits enables the construction of a complete set of logic circuit families and further realizes the design of decoders and shift registers.^270^ Iontronic logic circuits are also promising for multiple processing and computing functions. Feng *et al.* developed all carbon p–n-type ionic diodes and constructed simple processing units (AND and OR logic gates) for voltage-input computation.^210^ Similar AND and OR logic circuits have also been obtained *via* carbon-based capacitive ionic diodes (CAPodes) in Kaskel's team.^23^ Peng *et al.* also realized AND and OR logic gates *via* charge-modified CNT bipolar ion channels (Fig. 11f–i).^212^ Moreover, ionic diode-based logic circuits and ionic transistors have also been explored for NOT, NAND, and NOR logic computation. Cunha *et al.* developed a flexible and affordable smart iontronic logic circuit based on a fully screen-printed carbon-based ionic transistor.^267^ Afterward, low-voltage logic circuits have been successfully demonstrated in a millimeter-scale flexible iontronic system (Fig. 11j). Choi *et al.* further proposed CNT-based thin film ionic transistors which were further integrated into microscale flexible iontronic devices with logic circuit functions.^271^ NOT, NAND, and NOR logic gates have been constructed and the logic truth table of such logic gates has been confirmed in a microscale flexible system. The logic computation *via* carbon-based iontronics is summarized in Table 4.

**Table 4:** Summary and carbon-based ionic logic computing

Type of iontronics	Iontronic structures	Logic computing	Reference
Diode	Graphene oxides with functionalized positive (p) and negative (n) charges	AND, OR	210
Diode	Porous carbon-based capacitive ionic diodes (CAPodes)	AND, OR	23
Diode	MWCNTs decorated with oppositely charged polyelectrolytes on the channel entrance	AND, OR	212
Transistor	Screen-printed carbon-based ionic transistor with a semiconductor channel	NOT, NAND, NOR	267
Transistor	CNT thin-film transistors	NOT, NAND, NOR	271

The above-described iontronic logic computing systems have shown great potential for flexible and integrated devices, to simplify processing units for intelligent robotics, and to construct a smart “brain” for the internet of things. However, these ionic diode- and ionic transistor-based logic circuits still do not show significant advantages compared to conventional electronic logic gates, and still cannot deal with complex and dynamic issues, due to the lack of dynamic learning, and memorizing ability.

Kim *et al.* developed a CNT synaptic ionic transistor, which has demonstrated the dynamic logic, memory, and learning features of such a CNT synapse.^268^ In such a transistor-like CNT synapse, the spike from the presynaptic neuron triggered the current of the postsynaptic neuron (Fig. 11k). And two spatiotemporally correlated spikes on two CNT synapses induced two excitatory post-synaptic currents (EPSCs) with different amplitudes, which were summed in the post-synaptic neuron (Fig. 11l). The different EPSCs triggered by spatiotemporally correlated spikes showed the behavior of dynamic analog function of time and an inter-spike interval of two spikes (Fig. 11m). Therefore, the CNT synapse can also realize long-term potentiation (LTP) or depression (LTD) for memory features and spike-timing-dependent plasticity (STDP) with dynamic learning characteristics.^268^ Based on carbon materials, ionic logic computing systems can be an addition to and lead to enrichment of the existing electronics, and they can be integrated as a smart brain to interface with complex ionic molecules carrying chemical information in the future.

### 7.3. Actuating

After environmental stimuli are collected and processed in the sensing and processing units, the produced signals are further conducted in the actuating units. Biology relies on actuating components to adapt to complex environments, such as the release of pheromones,^272^ the contraction of the muscle system,^22^ and the rotation of the flagella.^273^ Carbon-based ionic pumps and ionic actuators have been widely developed to realize biomimetic functions, such as targeted ion delivery,^176^ bending, and rotating motions in recent years.^88,274^ Under an external electric field, targeted ions, such as small metallic ions, or DNA macromolecules, can be selectively delivered through artificial CNT ionic pumps, which can mimic the delivery and release of ions with specific chemical information.^71,275^

The biomimetic flagellum of paramecia has been realized by torsional carbon nanotube yarn, which is also powered by an applied voltage. These capacitive ionic rotatory motors overcame the problems of conventional rotary motors with complex and large structures.^45,236,244^ With their special rotating motion powered by electric signals, CNT-based rotators have shown promising potential for the simplification, miniaturization, and integration of actuating units in the “smart” iontronics.

**Fig. 12:**
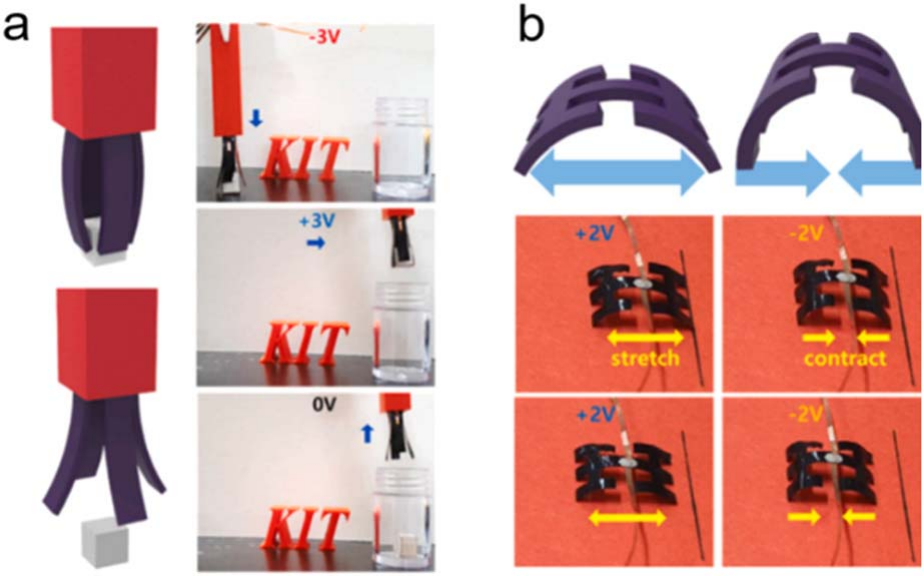
(a) Ionic bending actuator for bionic gripper and (b) soft locomotive robots. Reproduced with permission.^276^ Copyright 2023, Elsevier.

In addition, the bending movements such as bionic gripper and locomotive movements, can be mimicked by the carbon-based ionic actuator under an external voltage, as shown in Fig. 12a and b.^31,276^ Voltage-driven ionic actuating devices have simple structures and high energy-conversion efficiency, making them highly suitable for further actuating and integrating operations.^31^ Notably, the millimeter displacement of bent actuators needs around 3–5 s to reach the maximum values, which requires a second pulse of voltage.^276^ This means that action potentials from processing units need additional modulators or power sources to generate voltage pulses for powering these ionic actuators.^277^

### 7.4. Integrated artificial reflex arc

**Fig. 13:**
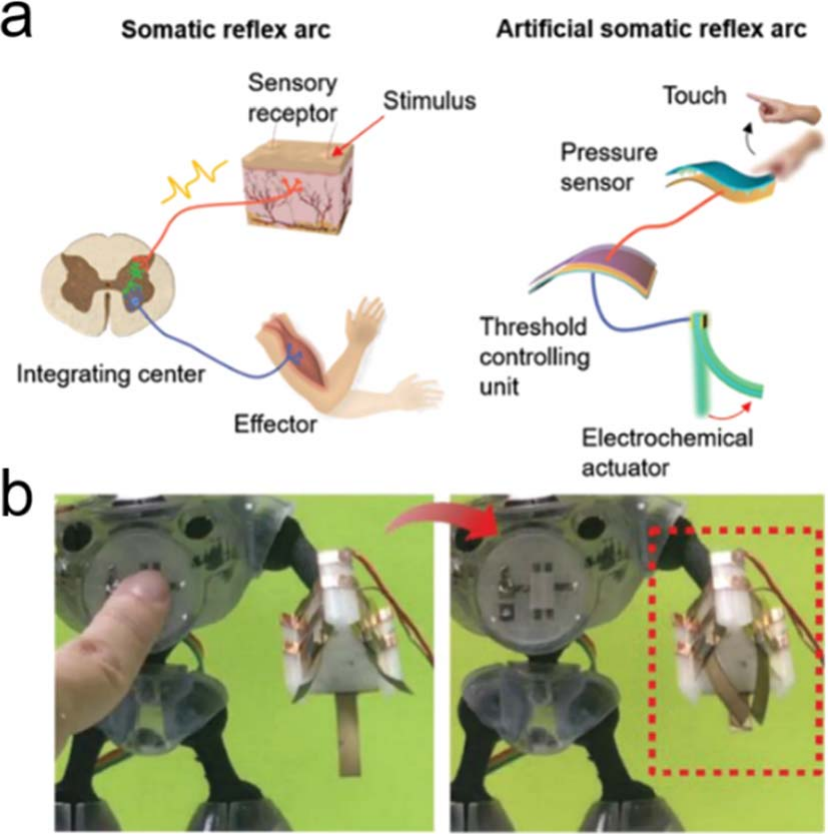
(a) The schematic comparison of the somatic reflex arc and the artificial somatic reflex arc, which consists of a pressure sensor, a threshold controlling unit (TCU), and an ionic actuator. (b) Photos of the robot hand responding to a firm tapping, where the artificial ionic actuator-based fingers tighten and grasp with a pressure level above the threshold. Reproduced with permission.^277^ Copyright 2019, John Wiley and Sons.

Differing from established centralized and sequential von Neumann computing structures, biological neural systems rely on decentralized, parallel, and event-driven computing structures to respond to multiple environmental stimuli and process parallel information in a highly efficient manner with ultralow energy consumption.^278,279^ The reflex arc as a basic structure of such biological information processing systems can be constructed by combining sensing, processing, and actuating compounds, which are promising for integrated artificial intelligent beings for mimicking multiple biological functions (Fig. 13).^277^

In the previous section, we have introduced and summarized the basic elements of carbon-based iontronics, including sensing (ionic capacitive pressure sensors, ionic transistors, and ionic diodes), processing (ionic transistors and ionic diodes), and actuating (ionic pumps and ionic actuators), which holds potential for an integrated artificial reflex arc. However, the mismatch and non-compatibility of the signals that are generated or received in these basic carbon-based iontronics are discouraging researchers from building highly integrated artificial reflex arcs. For example, the capacitance changes in ionic capacitive pressure sensors cannot be easily detected and processed by the processing unit (*i.e.*, logic computing systems composed of ionic transistors or diodes); the action potentials produced by processing units are insufficient to drive the ionic capacitive actuators due to the requirement of efficient current flows to drive ions and form EDLs. Fortunately, there are still many achievements in signal matching and compatibility in separated sensing, processing, and actuating units. Kim *et al.* proposed a flexible artificial afferent nerve to mimic a biological sensory nerve, which detects pressure signals *via* clusters of pressure sensors, converts them into action potentials *via* electronic ring oscillators, and further processes action potentials in a synaptic transistor.^280^ These afferent-nerve-like functions show a very high level of integration with no association with the additional instrument. The produced action potentials are further transmitted to the motor nerves of a detached cockroach leg and control the movement of the cockroach leg.

Furthermore, Chen *et al.* combined neurotransmitter biosensing, processing, and releasing to mimic the communication of neurons.^281^ However, all these elements are not carbon-based iontronics yet. The achievement of a reflex arc consisting of carbon-based iontronics was realized by Chen's group.^277^ They proposed an artificial somatic reflex arc, consisting of a pressure sensor unit, a threshold controlling unit, and an ionic actuator unit (Fig. 13a). The resistance of such a pressure sensor responds to the applied pressures; the resistance is high under weak pressure and linearly decreases with increasing pressure. When the resistance of such a pressure sensor is lower than the threshold value (*i.e.*, the pressure is higher than the threshold pressure), there is a very high current density in the circuit, activating the threshold controlling unit (composed of a ZIF-8-based ionic memristor^282^). The commercial reed switch is thereby activated and triggers the MWCNT/MnO_2_-based ionic actuators to move. The artificial ionic actuator-based fingers tighten and grasp with a pressure level above the threshold in a robot (Fig. 13b). Although such an iontronic reflex arc still needs some commercial electronics and additional power sources, it is a huge step toward a highly integrated reflex arc composed of carbon-based iontronics.^277^

## 8. Conclusions

In this review, we highlight recent advancements in the emerging field of carbon-based iontronics made of four distinct categories of carbon materials (fullerenes, CNTs, graphene, and porous carbons) to mimic biological processes, including sensing, processing, and actuating. The fundamental ion-electron coupling processes in these carbon-based iontronics are classified into three general types, charge transfer (*i.e.*, faradaic process), charge storage (*i.e.*, non-faradaic process), and charge interaction (*i.e.*, Coulomb drag process).

Carbon materials have seen great advancements and applications in the last few decades, especially in the fields of energy, environment, medicine, and electronics. Over recent years, the application of carbon materials in the iontronics field has attracted increasing attention, mainly because of the excellent performance of carbon materials, such as good electronic conductivity, chemical/electrochemical stability, ease of processing, and fine biocompatibility.

We thereby highlight five fundamental elements of carbon-based iontronics, ionic capacitive pressure sensors, ionic diodes, ionic transistors, ionic pumps, and ionic actuators, and summarize their basic bioinspired functions, such as sensing (ionic capacitive pressure sensors, ionic diodes, and ionic transistors), processing (ionic diodes and ionic transistors), and actuating (ionic pumps and ionic actuators). Currently, excellent biological functions are realized in devices, ranging from pressure sensing (ionic capacitive pressure sensors), biosensing (ionic transistors), logic and neuromorphic computing (ionic diodes and ionic transistors), and transmitter delivery and release (ionic pumps), to rotational/bending actuation (ionic actuators). These basic applications are mainly relying on single carbon-based iontronic elements without further integration, which is still far from a complex yet efficient biological process.

In biology, neural systems respond to multiple environmental stimuli relying on decentralized and parallel computing systems with an ultralow energy consumption, compared to traditional centralized and sequential computing structures. These decentralized and parallel information processing systems rely on the combinations of sensing, processing, and actuating compounds (*i.e.*, reflex arc), which integrate several basic biological functions to realize some specific functions. Consequently, the integration of these basic carbon-based iontronics holds potential for artificial intelligent beings, such as smart soft robots, interactive wearable devices, and neuroprosthetics. The main issues and obstacles currently lie in the fact that single-function devices have not considered the demands for integration into applications, which we call the mismatch and non-compatibility of elemental iontronic devices. Each of these iontronic elements requires the assistance of additional measurement instruments and power sources. The signal obtained from sensors (such as capacitance changes) cannot be transmitted and processed directly by the processing units. Also, the processing units cannot produce the required current or voltage to drive the actuating compounds. Therefore, deliberating on the basic design of fundamental carbon-based iontronic devices that match each other is paramount to fostering the evolution of highly integrated next-generation carbon-based iontronics in the future.

## Data availability

There are no raw data associated with this review.

## Author contributions

Panlong Li: investigation; writing – original draft; visualization of the sections of introduction, ion-related biological processes, electrolytes, carbon iontronics, biomimetic application, conclusions; review & editing of the whole manuscript. Przemyslaw Galek: investigation, writing – original draft and visualization of the sections of carbon materials and iontronics fabrication; review & editing of the whole manuscript. Julia Grothe: writing – review & editing; supervision. Stefan Kaskel: funding acquisition; writing – review & editing; supervision.

## Conflicts of interest

There are no conflicts to declare.

## References

[cit1] Bisri S. Z., Shimizu S., Nakano M., Iwasa Y. (2017). Adv. Mater..

[cit2] Mehonic A., Kenyon A. J. (2022). Nature.

[cit3] Masanet E., Shehabi A., Lei N., Smith S., Koomey J. (2020). Science.

[cit4] Istrate R., Tulus V., Grass R. N., Vanbever L., Stark W. J., Guillén-Gosálbez G. (2024). Nat. Commun..

[cit5] Amjadi M., Kyung K.-U., Park I., Sitti M. (2016). Adv. Funct. Mater..

[cit6] Sordan R., Traversi F., Russo V. (2009). Appl. Phys. Lett..

[cit7] Schwierz F. (2010). Nat. Nanotechnol..

[cit8] Li Q., Tian Z., Zhang X., Singh R., Du L., Gu J., Han J., Zhang W. (2015). Nat. Commun..

[cit9] Mohith S., Upadhya A. R., Navin K. P., Kulkarni S. M., Rao M. (2021). Smart Mater. Struct..

[cit10] Wan C., Xiao K., Angelin A., Antonietti M., Chen X. (2019). Adv. Intell. Syst..

[cit11] Chang Y., Wang L., Li R., Zhang Z., Wang Q., Yang J., Guo C. F., Pan T. (2021). Adv. Mater..

[cit12] Price A. S., Savchenko A. K., Narozhny B. N., Allison G., Ritchie D. A. (2007). Science.

[cit13] Simon D. T., Gabrielsson E. O., Tybrandt K., Berggren M. (2016). Chem. Rev..

[cit14] Rome L. C. (2006). Annu. Rev. Physiol..

[cit15] Jin P., Jan L. Y., Jan Y.-N. (2020). Annu. Rev. Neurosci..

[cit16] Bakry R., Vallant R. M., Najam-ul-Haq M., Rainer M., Szabo Z., Huck C. W., Bonn G. K. (2007). Int. J. Nanomed..

[cit17] Paradise M., Goswami T. (2007). Mater. Des..

[cit18] Zhu Y., Ji H., Cheng H.-M., Ruoff R. S. (2018). Natl. Sci. Rev..

[cit19] Li B., Xiong H., Xiao Y. (2020). Int. J. Electrochem. Sci..

[cit20] Li P., Bräuniger Y., Kunigkeit J., Zhou H., Ortega Vega M. R., Zhang E., Grothe J., Brunner E., Kaskel S. (2022). Angew. Chem., Int. Ed..

[cit21] Bräuniger Y., Lochmann S., Grothe J., Hantusch M., Kaskel S. (2021). ACS Appl. Energy Mater..

[cit22] Li Y., Liu W., Gao X., Zou T., Deng P., Zhao J., Zhang T., Chen Y., He L., Shao L., Yan Z., Zhang X. (2023). Sens. Actuators, A.

[cit23] Zhou H., Li P., Zhang E., Kunigkeit J., Zhou X., Haase K., Vega M. R. O., Wang S., Xu X., Grothe J. (2023). Angew. Chem..

[cit24] Huang P., Lethien C., Pinaud S., Brousse K., Laloo R., Turq V., Respaud M., Demortière A., Daffos B., Taberna P. L., Chaudret B., Gogotsi Y., Simon P. (2016). Science.

[cit25] Shen C., Wang X., Li S., Wang J., Zhang W., Kang F. (2013). J. Power Sources.

[cit26] Xu J., Shen G. (2015). Nano Energy.

[cit27] Zhao J., Guo H., Liu H., Fu T., Zhou W., Zhu Z., Hu Q. (2023). ACS Appl. Mater. Interfaces.

[cit28] Peng R., Pan Y., Liu B., Li Z., Pan P., Zhang S., Qin Z., Wheeler A. R., (Shirley) Tang X., Liu X. (2021). Small.

[cit29] Li X., Liu Y., Zhang J., Wu F., Hu M., Yang H. (2022). Adv. Intell. Syst..

[cit30] Rabinowitz J., Cohen C., Shepard K. L. (2020). Nano Lett..

[cit31] Kotal M., Kim J., Tabassian R., Roy S., Nguyen V. H., Koratkar N., Oh I.-K. (2018). Adv. Funct. Mater..

[cit32] Xiong Y., Han J., Wang Y., Wang Z. L., Sun Q. (2023). Research.

[cit33] Tybrandt K., Forchheimer R., Berggren M. (2012). Nat. Commun..

[cit34] Ren L., Li B., Wei G., Wang K., Song Z., Wei Y., Ren L., Liu Q. (2021). iScience.

[cit35] Martinac B. (2004). J. Cell Sci..

[cit36] Rayment I., Holden H. M., Whittaker M., Yohn C. B., Lorenz M., Holmes K. C., Milligan R. A. (1993). Science.

[cit37] Guan L., Yu L., Chen G. Z. (2016). Electrochim. Acta.

[cit38] Zhang E., Galle L., Lochmann S., Grothe J., Kaskel S. (2021). Chem. Eng. J..

[cit39] Li P., Fang Z., Dong X., Wang C., Xia Y. (2022). Natl. Sci. Rev..

[cit40] Béguin F., Presser V., Balducci A., Frackowiak E. (2014). Adv. Mater..

[cit41] Xu F., Zhai Y., Zhang E., Liu Q., Jiang G., Xu X., Qiu Y., Liu X., Wang H., Kaskel S. (2020). Angew. Chem..

[cit42] Narozhny B. N., Levchenko A. (2016). Rev. Mod. Phys..

[cit43] Bischak C. G., Flagg L. Q., Yan K., Li C.-Z., Ginger D. S. (2019). ACS Appl. Mater. Interfaces.

[cit44] Ye J., Craciun M. F., Koshino M., Russo S., Inoue S., Yuan H., Shimotani H., Morpurgo A. F., Iwasa Y. (2011). Proc. Natl. Acad. Sci. U. S. A..

[cit45] Foroughi J., Spinks G. M., Wallace G. G., Oh J., Kozlov M. E., Fang S., Mirfakhrai T., Madden J. D. W., Shin M. K., Kim S. J., Baughman R. H. (2011). Science.

[cit46] Astefanei A., Núñez O., Galceran M. T. (2015). Anal. Chim. Acta.

[cit47] Rathinavel S., Priyadharshini K., Panda D. (2021). Mater. Sci. Eng. B.

[cit48] Allen M. J., Tung V. C., Kaner R. B. (2010). Chem. Rev..

[cit49] Tian W., Zhang H., Duan X., Sun H., Shao G., Wang S. (2020). Adv. Funct. Mater..

[cit50] Goodarzi S., Da Ros T., Conde J., Sefat F., Mozafari M. (2017). Mater. Today.

[cit51] Nasir S., Hussein M. Z., Zainal Z., Yusof N. A. (2018). Materials.

[cit52] Aqel A., El-Nour K. M. M. A., Ammar R. A. A., Al-Warthan A. (2012). Arab. J. Chem..

[cit53] Slanina Z. (2001). NTM Int. J. Hist. Ethics Nat. Sci. Technol. Med..

[cit54] Dinadayalane T. C., Leszczynski J. (2010). Struct. Chem..

[cit55] Sariciftci N. S., Smilowitz L., Heeger A. J., Wudl F. (1992). Science.

[cit56] Ganesamoorthy R., Sathiyan G., Sakthivel P. (2017). Sol. Energy Mater. Sol. Cells.

[cit57] Hou J., Inganäs O., Friend R. H., Gao F. (2018). Nat. Mater..

[cit58] Gatti T., Menna E., Meneghetti M., Maggini M., Petrozza A., Lamberti F. (2017). Nano Energy.

[cit59] Chen Y., Wu H., Jin F., Ge H.-L., Gao F., Wu Q., Wang S., Wang Y., Yang H. (2024). J. Colloid Interface Sci..

[cit60] Zhao M., Wang C., Ji C., Liu R., Xie J., Wang Y., Gu Z. (2023). Small.

[cit61] Wang C., Zhao M., Xie J., Wang H., Gu Z., Sun F. (2023). Adv. Healthcare Mater..

[cit62] Wang C., Zhao M., Xie J., Ji C., Leng Z., Gu Z. (2022). Chem. Eng. J..

[cit63] Halenova T. I., Vareniuk I. M., Roslova N. M., Dzerzhynsky M. E., Savchuk O. M., Ostapchenko L. I., Prylutskyy Yu. I., Ritter U., Scharff P. (2016). RSC Adv..

[cit64] Gonchar O. O., V Maznychenko A., V Bulgakova N., V Vereshchaka I., Tomiak T., Ritter U., Prylutskyy Y. I., Mankovska I. M., Kostyukov A. I. (2018). Oxid. Med. Cell. Longevity.

[cit65] Xiao L., Takada H., hui Gan X., Miwa N. (2006). Bioorg. Med. Chem. Lett..

[cit66] Narayan R. , Nanobiomaterials: nanostructured materials for biomedical applications, Woodhead Publishing, 2017

[cit67] De Jong W. H., Borm P. J. A. (2008). Int. J. Nanomed..

[cit68] Prylutska S., Grynyuk I., Skaterna T., Horak I., Grebinyk A., Drobot L., Matyshevska O., Senenko A., Prylutskyy Y., Naumovets A., Ritter U., Frohme M. (2019). Arch. Toxicol..

[cit69] Rabinowitz J., Cohen C., Shepard K. L. (2020). Nano Lett..

[cit70] Rashed A. O., Merenda A., Kondo T., Lima M., Razal J., Kong L., Huynh C., Dumée L. F. (2021). Sep. Purif. Technol..

[cit71] Geng J., Kim K., Zhang J., Escalada A., Tunuguntla R., Comolli L. R., Allen F. I., V Shnyrova A., Cho K. R., Munoz D., Wang Y. M., Grigoropoulos C. P., Ajo-Franklin C. M., Frolov V. A., Noy A. (2014). Nature.

[cit72] Zhou H., Li W., Yu P. (2022). ChemistryOpen.

[cit73] Liu H., Nishide D., Tanaka T., Kataura H. (2011). Nat. Commun..

[cit74] Krupke R., Hennrich F., Löhneysen H. v., Kappes M. M. (2003). Science.

[cit75] Rother M., Kruse A., Brohmann M., Matthiesen M., Grieger S., Higgins T. M., Zaumseil J. (2018). ACS Appl. Nano Mater..

[cit76] Baughman R. H., Cui C., Zakhidov A. A., Iqbal Z., Barisci J. N., Spinks G. M., Wallace G. G., Mazzoldi A., De Rossi D., Rinzler A. G., Jaschinski O., Roth S., Kertesz M. (1999). Science.

[cit77] Hyeon J. S., Park J. W., Baughman R. H., Kim S. J. (2019). Sens. Actuators, B.

[cit78] Liu J., Gao Y., Wang H., Poling-Skutvik R., Osuji C. O., Yang S. (2020). Adv. Intell. Syst..

[cit79] Kim H., Lee J. A., Ambulo C. P., Lee H. B., Kim S. H., V Naik V., Haines C. S., Aliev A. E., Ovalle-Robles R., Baughman R. H., Ware T. H. (2019). Adv. Funct. Mater..

[cit80] Bianco A., Kostarelos K., Prato M. (2011). Chem. Commun..

[cit81] Gooding J. J., Wibowo R., Liu J., Yang W., Losic D., Orbons S., Mearns F. J., Shapter J. G., Hibbert D. B. (2003). J. Am. Chem. Soc..

[cit82] Yu X., Munge B., Patel V., Jensen G., Bhirde A., Gong J. D., Kim S. N., Gillespie J., Gutkind J. S., Papadimitrakopoulos F., Rusling J. F. (2006). J. Am. Chem. Soc..

[cit83] He P., Li S., Dai L. (2005). Synth. Met..

[cit84] Geim A. K., Novoselov K. S. (2007). Nat. Mater..

[cit85] Peres N. M. R., Ribeiro R. M. (2009). New J. Phys..

[cit86] Zdetsis A. D., Economou E. N. (2015). J. Phys. Chem. C.

[cit87] Novoselov K. S., Geim A. K., V Morozov S., Jiang D., Zhang Y., V Dubonos S., V Grigorieva I., Firsov A. A. (2004). Science.

[cit88] Wang B., Huang P., Li B., Wu Z., Xing Y., Zhu J., Liu L. (2023). Small.

[cit89] Wang X., Lim E. G., Hoettges K., Song P. (2023). C-J. Carbon Res..

[cit90] Verma A. M., Honkala K., Melander M. M. (2021). Front. Energy Res..

[cit91] Dong S., Zhang Y., Li B., Ren J., Ling S., Cao L. (2021). Nanotechnology.

[cit92] Zhang Z., Kong X.-Y., Xiao K., Liu Q., Xie G., Li P., Ma J., Tian Y., Wen L., Jiang L. (2015). J. Am. Chem. Soc..

[cit93] Zhang X., Wen Q., Wang L., Ding L., Yang J., Ji D., Zhang Y., Jiang L., Guo W. (2019). ACS Nano.

[cit94] Novodchuk I., Bajcsy M., Yavuz M. (2021). Carbon.

[cit95] Fic K., Platek A., Piwek J., Frackowiak E. (2018). Mater. Today.

[cit96] Lee J., Kim J., Hyeon T. (2006). Adv. Mater..

[cit97] Beck J. S., Vartuli J. C., Roth W. J., Leonowicz M. E., Kresge C. T., Schmitt K. D., Chu C. T. W., Olson D. H., Sheppard E. W., McCullen S. B., Higgins J. B., Schlenker J. L. (1992). J. Am. Chem. Soc..

[cit98] Ryoo R., Joo S. H., Kruk M., Jaroniec M. (2001). Adv. Mater..

[cit99] Li P., Unglaube N., Zhou H., Michel S., Dong X., Xu X., Birnbaum A., Auernhammer G. K., Xia Y., Grothe J., Kaskel S. (2023). Chem. Eng. J..

[cit100] Shao H., Wu Y.-C., Lin Z., Taberna P.-L., Simon P. (2020). Chem. Soc. Rev..

[cit101] Yang Z., Cao S., Lv T., Zhang G., Guo X., Zhang S., Pang H. (2022). MRS Energy Sustain..

[cit102] Sayago J., Soavi F., Sivalingam Y., Cicoira F., Santato C. (2014). J. Mater. Chem. C.

[cit103] Tang H., Kumar P., Zhang S., Yi Z., De Crescenzo G., Santato C., Soavi F., Cicoira F. (2015). ACS Appl. Mater. Interfaces.

[cit104] Supiyeva Z., Pan X., Abbas Q. (2023). Curr. Opin. Electrochem..

[cit105] Fuertes A. B., Ferrero G. A., Sevilla M. (2014). J. Mater. Chem. A.

[cit106] Saha D., Heldt C. L., Gencoglu M. F., Vijayaragavan K. S., Chen J., Saksule A. (2016). Mater. Sci. Eng. C.

[cit107] Llamas-Unzueta R., Suárez M., Fernández A., Díaz R., Montes-Morán M. A., Menéndez J. A. (2021). Biomedicines.

[cit108] Vitus V., Ibrahim F., Wan Kamarul Zaman W. S. (2021). Polymers.

[cit109] Bharti C., Nagaich U., Pal A. K., Gulati N. (2015). Int. J. Pharm. Invest..

[cit110] Robert K., Douard C., Demortière A., Blanchard F., Roussel P., Brousse T., Lethien C. (2018). Adv. Mater. Technol..

[cit111] Ouendi S., Robert K., Stievenard D., Brousse T., Roussel P., Lethien C. (2019). Energy Storage Mater..

[cit112] Zhu D., Liu Y., Yuan L., Liu Y., Li X., Yi L., Wei H., Yao K. (2014). RSC Adv..

[cit113] Barranco V., Lillo-Rodenas M. A., Linares-Solano A., Oya A., Pico F., Ibañez J., Agullo-Rueda F., Amarilla J. M., Rojo J. M. (2010). J. Phys. Chem. C.

[cit114] Chen Y., Lu C. (2023). Carbon Neutralization.

[cit115] V Shishkovsky I. and Lebedev P. N., in Nanocoatings and Ultra-Thin Films, ed. A. S. H. Makhlouf and I. Tiginyanu, Woodhead Publishing, 2011, pp. 57

[cit116] Muratore C., Voevodin A. A., Glavin N. R. (2019). Thin Solid Films.

[cit117] Garlow J. A., Barrett L. K., Wu L., Kisslinger K., Zhu Y., Pulecio J. F. (2016). Sci. Rep..

[cit118] Dinh T. M., Mesnilgrente F., Conédéra V., Kyeremateng N. A., Pech D. (2015). J. Electrochem. Soc..

[cit119] Niu Z., Zhang L., Liu L., Zhu B., Dong H., Chen X. (2013). Adv. Mater..

[cit120] Soam A., Kavle P., Kumbhar A., Dusane R. O. (2017). Curr. Appl. Phys..

[cit121] Shen B., Lang J., Guo R., Zhang X., Yan X. (2015). ACS Appl. Mater. Interfaces.

[cit122] Huang P., Pech D., Lin R., McDonough J. K., Brunet M., Taberna P.-L., Gogotsi Y., Simon P. (2013). Electrochem. Commun..

[cit123] Jin E. M., Lee H. J., Jun H.-B., Jeong S. M. (2017). Korean J. Chem. Eng..

[cit124] Zhou J., Chen N., Ge Y., Zhu H., Feng X., Liu R., Ma Y., Wang L., Hou W. (2018). Sci. China Mater..

[cit125] Han C., Cao W., Si H., Wu Y., Liu K., Liu H., Sang S., Wu Q. (2019). Electrochim. Acta.

[cit126] Zhang P., Wang F., Yang S., Wang G., Yu M., Feng X. (2020). Energy Storage Mater..

[cit127] Zhao D., Chen C., Zhang Q., Chen W., Liu S., Wang Q., Liu Y., Li J., Yu H. (2017). Adv. Energy Mater..

[cit128] Yun J., Lim Y., Lee H., Lee G., Park H., Hong S. Y., Jin S. W., Lee Y. H., Lee S.-S., Ha J. S. (2017). Adv. Funct. Mater..

[cit129] Boruah B. D., Nandi S., Misra A. (2018). ACS Appl. Energy Mater..

[cit130] Jiang Q., Kurra N., Maleski K., Lei Y., Liang H., Zhang Y., Gogotsi Y., Alshareef H. N. (2019). Adv. Energy Mater..

[cit131] Kim S.-W., Kang K.-N., Min J.-W., Jang J.-H. (2018). Nano Energy.

[cit132] Wang S., Wu Z.-S., Zheng S., Zhou F., Sun C., Cheng H.-M., Bao X. (2017). ACS Nano.

[cit133] Boruah B. D., Maji A., Misra A. (2018). ACS Appl. Mater. Interfaces.

[cit134] Yin J., Kim J., Lee H. U., Park J. Y. (2018). Thin Solid Films.

[cit135] He Y., Zhang P., Wang F., Wang L., Su Y., Zhang F., Zhuang X., Feng X. (2019). Nano Energy.

[cit136] Tian W., VahidMohammadi A., Reid M. S., Wang Z., Ouyang L., Erlandsson J., Pettersson T., Wågberg L., Beidaghi M., Hamedi M. M. (2019). Adv. Mater..

[cit137] Zhou F., Huang H., Xiao C., Zheng S., Shi X., Qin J., Fu Q., Bao X., Feng X., Müllen K., Wu Z.-S. (2018). J. Am. Chem. Soc..

[cit138] Zheng S., Ma J., Wu Z.-S., Zhou F., He Y.-B., Kang F., Cheng H.-M., Bao X. (2018). Energy Environ. Sci..

[cit139] Qin J., Wang S., Zhou F., Das P., Zheng S., Sun C., Bao X., Wu Z.-S. (2019). Energy Storage Mater..

[cit140] Liu Z., Liu S., Dong R., Yang S., Lu H., Narita A., Feng X., Müllen K. (2017). Small.

[cit141] Vyas A., Hajibagher S. Z., Méndez-Romero U., Thurakkal S., Li Q., Haque M., Azega R. K., Wang E., Zhang X., Lundgren P., Enoksson P., Smith A. (2022). ACS Appl. Energy Mater..

[cit142] Su S., Lai L., Li R., Lin Y., Dai H., Zhu X. (2020). ACS Appl. Energy Mater..

[cit143] Altin Y., Celik Bedeloglu A. (2021). J. Mater. Sci.: Mater. Electron..

[cit144] Zhu J., Qin Y., Zhang Y. (2009). Electrochem. Commun..

[cit145] Kaliyaraj Selva Kumar A., Zhang Y., Li D., Compton R. G. (2020). Electrochem. Commun..

[cit146] Zhang Y.-Z., Wang Y., Cheng T., Yao L.-Q., Li X., Lai W.-Y., Huang W. (2019). Chem. Soc. Rev..

[cit147] Li H., Liang J. (2020). Adv. Mater..

[cit148] Khan S., Lorenzelli L., Dahiya R. S. (2015). IEEE Sens. J..

[cit149] Hatala M., Gemeiner P., Hvojnik M., Mikula M. (2019). J. Mater. Sci.: Mater. Electron..

[cit150] Gao C., Gao J., Shao C., Xiao Y., Zhao Y., Qu L. (2018). J. Mater. Chem. A.

[cit151] Zhang C., Kremer M. P., Seral-Ascaso A., Park S.-H., McEvoy N., Anasori B., Gogotsi Y., Nicolosi V. (2018). Adv. Funct. Mater..

[cit152] Krebs F. C. (2009). Sol. Energy Mater. Sol. Cells.

[cit153] Grau G., Cen J., Kang H., Kitsomboonloha R., Scheideler W. J., Subramanian V. (2016). Flexible Printed Electron..

[cit154] Gao D., Wang J., Ai K., Xiong J., Li S., Lee P. S. (2020). Adv. Intell. Syst..

[cit155] Krzeminski J., Kanthamneni A., Wagner D., Detert M., Schmidt B., Jakubowska M. (2018). IEEE Trans. Nanotechnol..

[cit156] Wilkinson N. J., Smith M. A. A., Kay R. W., Harris R. A. (2019). Int. J. Adv. Manuf. Technol..

[cit157] Seifert T., Sowade E., Roscher F., Wiemer M., Gessner T., Baumann R. R. (2015). Ind. Eng. Chem. Res..

[cit158] Mahajan A., Frisbie C. D., Francis L. F. (2013). ACS Appl. Mater. Interfaces.

[cit159] Zhang Y.-Z., Wang Y., Cheng T., Yao L.-Q., Li X., Lai W.-Y., Huang W. (2019). Chem. Soc. Rev..

[cit160] Zhang F., Wei M., V Viswanathan V., Swart B., Shao Y., Wu G., Zhou C. (2017). Nano Energy.

[cit161] Chang P., Mei H., Zhou S., Dassios K. G., Cheng L. (2019). J. Mater. Chem. A.

[cit162] Tian X., Jin J., Yuan S., Chua C. K., Tor S. B., Zhou K. (2017). Adv. Energy Mater..

[cit163] Mohan V. B., Krebs B. J., Bhattacharyya D. (2018). Mater. Today Commun..

[cit164] Wiggins P. M. (1990). Microbiol. Rev..

[cit165] Dechiraju H., Jia M., Luo L., Rolandi M. (2022). Adv. Sustainable Syst..

[cit166] Choi S.-G., Kang S.-H., Lee J.-Y., Park J.-H., Kang S.-K. (2023). Front. Bioeng. Biotechnol..

[cit167] Elvira K. S., Gielen F., Tsai S. S. H., Nightingale A. M. (2022). Lab Chip.

[cit168] Lu L., Jiang C., Hu G., Liu J., Yang B. (2021). Adv. Mater..

[cit169] Wu H., Yang G., Zhu K., Liu S., Guo W., Jiang Z., Li Z. (2021). Adv. Sci..

[cit170] Lochmann S., Bräuniger Y., Gottsmann V., Galle L., Grothe J., Kaskel S. (2020). Adv. Funct. Mater..

[cit171] Bräuniger Y., Lochmann S., Gellrich C., Galle L., Grothe J., Kaskel S. (2022). Energy Technol..

[cit172] Zhang E., Fulik N., Hao G.-P., Zhang H.-Y., Kaneko K., Borchardt L., Brunner E., Kaskel S. (2019). Angew. Chem., Int. Ed..

[cit173] Bin D., Du Y., Yang B., Lu H., Liu Y., Xia Y. (2023). Adv. Funct. Mater..

[cit174] Dong W., Lin T., Huang J., Wang Y., Zhang Z., Wang X., Yuan X., Lin J., Chen I.-W., Huang F. (2020). Research.

[cit175] Li P., Feder-Kubis J., Kunigkeit J., Zielińska-Błajet M., Brunner E., Grothe J., Kaskel S. (2024). Angew. Chem., Int. Ed..

[cit176] Sjostrom T. A., Berggren M., Gabrielsson E. O., Janson P., Poxson D. J., Seitanidou M., Simon D. T. (2018). Adv. Mater. Technol..

[cit177] Li P., Tarasova A., Slesinski A., Bräuniger Y., Galek P., Grothe J., Kaskel S. (2024). Adv. Mater. Technol..

[cit178] Zhang P., Wang K., Pei P., Zuo Y., Wei M., Liu X., Xiao Y., Xiong J. (2021). Mater. Today Chem..

[cit179] Zhang P., Guo W., Guo Z. H., Ma Y., Gao L., Cong Z., Zhao X. J., Qiao L., Pu X., Wang Z. L. (2021). Adv. Mater..

[cit180] Guo L., Ma W.-B., Wang Y., Song X.-Z., Ma J., Han X.-D., Tao X.-Y., Guo L.-T., Fan H.-L., Liu Z.-S., Zhu Y.-B., Wei X.-Y. (2020). J. Alloys Compd..

[cit181] Ye Y., Zhang Y., Chen Y., Han X., Jiang F. (2020). Adv. Funct. Mater..

[cit182] Shen Z., Ma J., Cai Y., Li S., Ruan D., Dai S., Sheng Z., Bai J., Yin D., Ping J., Ying Y., Yang C., Qu S., Jia Z. (2023). Cell Rep. Phys. Sci..

[cit183] Keplinger C., Sun J.-Y., Foo C. C., Rothemund P., Whitesides G. M., Suo Z. (2013). Science.

[cit184] Cheng C.-Y., Pai W.-L., Chen Y.-H., Paylaga N. T., Wu P.-Y., Chen C.-W., Liang C.-T., Chou F.-C., Sankar R., Fuhrer M. S., Chen S.-Y., Wang W.-H. (2022). Nano Lett..

[cit185] Nketia-Yawson B., Ahn H., Jo J. W. (2022). Adv. Funct. Mater..

[cit186] Lee G., Kim D., Kim D., Oh S., Yun J., Kim J., Lee S.-S., Ha J. S. (2015). Energy Environ. Sci..

[cit187] Fuller E. J., El Gabaly F., Léonard F., Agarwal S., Plimpton S. J., Jacobs-Gedrim R. B., James C. D., Marinella M. J., Talin A. A. (2017). Adv. Mater..

[cit188] Hallinan D. T., Balsara N. P. (2013). Annu. Rev. Mater. Res..

[cit189] Feng W., Dong X., Zhang X., Lai Z., Li P., Wang C., Wang Y., Xia Y. (2020). Angew. Chem., Int. Ed..

[cit190] Feng W., Dong X., Li P., Wang Y., Xia Y. (2019). J. Power Sources.

[cit191] Kim S. J., Kim S., Jang H. W. (2021). iScience.

[cit192] Xiong T., Li C., He X., Xie B., Zong J., Jiang Y., Ma W., Wu F., Fei J., Yu P., Mao L. (2023). Science.

[cit193] Khalid M. (2019). Trans. Electr. Electron. Mater..

[cit194] Chen W., Yan X. (2020). J. Mater. Sci. Technol..

[cit195] Salanne M., Rotenberg B., Naoi K., Kaneko K., Taberna P.-L., Grey C. P., Dunn B., Simon P. (2016). Nat. Energy.

[cit196] Forse A. C., Merlet C., Griffin J. M., Grey C. P. (2016). J. Am. Chem. Soc..

[cit197] Mishra R. B., El-Atab N., Hussain A. M., Hussain M. M. (2021). Adv. Mater. Technol..

[cit198] Huang Y., Fan X., Chen S.-C., Zhao N. (2019). Adv. Funct. Mater..

[cit199] Kwon D., Lee T.-I., Shim J., Ryu S., Kim M. S., Kim S., Kim T.-S., Park I. (2016). ACS Appl. Mater. Interfaces.

[cit200] Wang H., Li Z., Liu Z., Fu J., Shan T., Yang X., Lei Q., Yang Y., Li D. (2022). J. Mater. Chem. C.

[cit201] Wei N., Li Y., Tang Y., Zhou Y., Ning R., Tang M., Lu S., Zeng W., Xiong Y. (2022). J. Colloid Interface Sci..

[cit202] Ma R., Zhao Y., Chen H., Zeng Z., Li Y., Wang R., Liu F. (2024). Adv. Eng. Mater..

[cit203] Yoon H., Ko S., Chhetry A., Park C., Sharma S., Yoon S., Kim D., Zhang S., Kim D. H., Park J. Y. (2022). Adv. Mater. Technol..

[cit204] Sharma S., Pradhan G. B., Chhetry A., Shrestha K., Bhatta T., Zhang S., Kim D., Jeong S., Shin Y., Zahed Md. A., Hui X., Park J. Y. (2023). Nano Today.

[cit205] Cicek M. O., Durukan M. B., Yıldız B., Keskin D., Doganay D., Çınar Aygün S., Cakir M. P., Unalan H. E. (2023). Adv. Mater. Technol..

[cit206] Yoon H., Ko S., Chhetry A., Park C., Sharma S., Yoon S., Kim D., Zhang S., Kim D. H., Park J. Y. (2022). Adv. Mater. Technol..

[cit207] Nguyen G., Vlassiouk I., Siwy Z. S. (2010). Nanotechnology.

[cit208] Huang X., Kong X.-Y., Wen L., Jiang L. (2018). Adv. Funct. Mater..

[cit209] Gao J., Guo W., Feng D., Wang H., Zhao D., Jiang L. (2014). J. Am. Chem. Soc..

[cit210] Feng X., Zhao X., Yang L., Li M., Qie F., Guo J., Zhang Y., Li T., Yuan W., Yan Y. (2018). Nat. Commun..

[cit211] Li S., Zhang X., Su J. (2023). J. Phys. Chem. C.

[cit212] Peng R., Pan Y., Li Z., Zhang S., Wheeler A. R., Tang X., Liu X. (2020). Adv. Funct. Mater..

[cit213] Gouaux E., MacKinnon R. (2005). Science.

[cit214] Hao J., Sun Q., Wang W., Zhao M., Liu H., Sui X. (2023). J. Power Sources.

[cit215] Xiao K., Wen L., Jiang L. (2016). Small.

[cit216] Kim J., Jeon J., Wang C., Chang G. T., Park J. (2022). ACS Nano.

[cit217] Li M., Wang C., Liu Z., Song Y., Li D. (2021). Adv. Funct. Mater..

[cit218] Mathew M., V Shinde P., Samal R., Rout C. S. (2021). J. Mater. Sci..

[cit219] Feng J., Wang Y., Xu Y., Ma H., Wang G., Ma P., Tang Y., Yan X. (2021). Adv. Mater..

[cit220] Uesugi E., Goto H., Eguchi R., Fujiwara A., Kubozono Y. (2013). Sci. Rep..

[cit221] Prakoso S. P., Chen M.-N., Chiu Y.-C. (2022). J. Mater. Chem. C.

[cit222] Shimotani H., Kanbara T., Iwasa Y., Tsukagoshi K., Aoyagi Y., Kataura H. (2006). Appl. Phys. Lett..

[cit223] Xu F., Wu M.-Y., Safron N. S., Roy S. S., Jacobberger R. M., Bindl D. J., Seo J.-H., Chang T.-H., Ma Z., Arnold M. S. (2014). Nano Lett..

[cit224] Li H., Zhou L. (2015). ACS Appl. Mater. Interfaces.

[cit225] Fakih I., Durnan O., Mahvash F., Napal I., Centeno A., Zurutuza A., Yargeau V., Szkopek T. (2020). Nat. Commun..

[cit226] Chen F., Zhao Y., Saxena A., Zhao C., Niu M., Aluru N. R., Feng J. (2023). Nano Lett..

[cit227] Gadsby D. C. (2009). Nat. Rev. Mol. Cell Biol..

[cit228] Seitanidou M., Blomgran R., Pushpamithran G., Berggren M., Simon D. T. (2019). Adv. Healthcare Mater..

[cit229] Isaksson J., Kjäll P., Nilsson D., Robinson N., Berggren M., Richter-Dahlfors A. (2007). Nat. Mater..

[cit230] Marcotte A., Mouterde T., Niguès A., Siria A., Bocquet L. (2020). Nat. Mater..

[cit231] Ma Q., Li Y., Wang R., Xu H., Du Q., Gao P., Xia F. (2021). Nat. Commun..

[cit232] Peng W., Yan S., Zhou K., Wu H.-C., Liu L., Zhao Y. (2023). Nat. Commun..

[cit233] Liu L., Yang C., Zhao K., Li J., Wu H.-C. (2013). Nat. Commun..

[cit234] He K., Wang C., He Y., Su J., Chen X. (2023). Chem. Rev..

[cit235] Kotal M., Tabassian R., Roy S., Oh S., Oh I.-K. (2020). Adv. Funct. Mater..

[cit236] Park C.-L., Goh B., Kim E. S., Choi J., Kim S. H. (2024). Carbon.

[cit237] Tabassian R., Kim J., Nguyen V. H., Kotal M., Oh I.-K. (2018). Adv. Funct. Mater..

[cit238] Ying Z., Wang Q., Xie J., Li B., Lin X., Hui S. (2020). J. Mater. Chem. C.

[cit239] Roy S., Kim J., Kotal M., Tabassian R., Kim K. J., Oh I.-K. (2019). Adv. Funct. Mater..

[cit240] Basheer A. A. (2023). Mater. Chem. Phys..

[cit241] Sun G., Kürti J., Kertesz M., Baughman R. H. (2002). J. Am. Chem. Soc..

[cit242] Sun G., Kertesz M., Kürti J., Baughman R. H. (2003). Phys. Rev. B:Condens. Matter Mater. Phys..

[cit243] Wang B., Huang P., Li B., Wu Z., Xing Y., Zhu J., Liu L. (2023). Small.

[cit244] Chu H., Hu X., Wang Z., Mu J., Li N., Zhou X., Fang S., Haines C. S., Park J. W., Qin S., Yuan N., Xu J., Tawfick S., Kim H., Conlin P., Cho M., Cho K., Oh J., Nielsen S., Alberto K. A., Razal J. M., Foroughi J., Spinks G. M., Kim S. J., Ding J., Leng J., Baughman R. H. (2021). Science.

[cit245] Chen Y., Zhang P., Li Y., Zhang K., Su J., Huang L. (2021). J. Phys. D Appl. Phys..

[cit246] Yao Y., Lu X., Fu C., Zhang Y., Fang J., Qin J., He Q.-C., Yang T. (2024). Adv. Funct. Mater..

[cit247] Kim J., Jeon J., Wang C., Chang G. T., Park J. (2022). ACS Nano.

[cit248] Bu X., Xu H., Shang D., Li Y., Lv H., Liu Q. (2020). Adv. Intell. Syst..

[cit249] Sabbagh B., Fraiman N. E., Fish A., Yossifon G. (2023). ACS Appl. Mater. Interfaces.

[cit250] Liu W., Mei T., Cao Z., Li C., Wu Y., Wang L., Xu G., Chen Y., Zhou Y., Wang S., Xue Y., Yu Y., Kong X.-Y., Chen R., Tu B., Xiao K. (2024). Sci. Adv..

[cit251] Huang C., Hao Z., Qi T., Pan Y., Zhao X. (2020). J. Materiomics.

[cit252] Zhang S., Guo J., Liu L., Ruan H., Kong C., Yuan X., Zhang B., Gu G., Cui P., Cheng G., Du Z. (2022). Nano Energy.

[cit253] Qureshi A., Niazi J. H. (2023). Mater. Horiz..

[cit254] Ma M., Zhou Y., Li J., Ge Z., He H., Tao T., Cai Z., Wang X., Chang G., He Y. (2020). Analyst.

[cit255] Tran T.-T., Mulchandani A. (2016). Trac. Trends Anal. Chem..

[cit256] Liao C., Mak C., Zhang M., Chan H. L. W., Yan F. (2015). Adv. Mater..

[cit257] Xiao K., Wan C., Jiang L., Chen X., Antonietti M. (2020). Adv. Mater..

[cit258] Dai S., Liu X., Liu Y., Xu Y., Zhang J., Wu Y., Cheng P., Xiong L., Huang J. (2023). Adv. Mater..

[cit259] Zhao C., Wang Y., Tang G., Ru J., Zhu Z., Li B., Guo C. F., Li L., Zhu D. (2022). Adv. Funct. Mater..

[cit260] Zhang H., Yu J., Yang X., Gao G., Qin S., Sun J., Ding M., Jia C., Sun Q., Wang Z. L. (2020). ACS Nano.

[cit261] Peng P., Yang F., Wang Z., Wei D. (2023). Adv. Energy Mater..

[cit262] Zhu P., Du H., Hou X., Lu P., Wang L., Huang J., Bai N., Wu Z., Fang N. X., Guo C. F. (2021). Nat. Commun..

[cit263] Parichenko A., Huang S., Pang J., Ibarlucea B., Cuniberti G. (2023). Trac. Trends Anal. Chem..

[cit264] Laleh S., Ibarlucea B., Stadtmüller M., Cuniberti G., Medina-Sánchez M. (2023). Biosens. Bioelectron..

[cit265] Wang J., Lin M.-F., Park S., Lee P. S. (2018). Mater. Today.

[cit266] van de Burgt Y., Lubberman E., Fuller E. J., Keene S. T., Faria G. C., Agarwal S., Marinella M. J., Alec Talin A., Salleo A. (2017). Nat. Mater..

[cit267] Cunha I., Ferreira S. H., Martins J., Fortunato E., Gaspar D., Martins R., Pereira L. (2022). Adv. Sustainable Syst..

[cit268] Kim K., Chen C.-L., Truong Q., Shen A. M., Chen Y. (2013). Adv. Mater..

[cit269] Tang P., Tan W., Li F., Xue S., Ma Y., Jing P., Liu Y., Zhu J., Yan X. (2023). Adv. Mater..

[cit270] Wang X.-Y., Dong C.-T., Wu Z.-R., Cheng Z.-Q. (2021). Chin. Phys. B.

[cit271] Choi Y., Kang J., Secor E. B., Sun J., Kim H., Lim J. A., Kang M. S., Hersam M. C., Cho J. H. (2018). Adv. Funct. Mater..

[cit272] Landaeta-Hernández A. J., Ungerfeld R., Chenoweth P. J. (2023). Anim. Reprod. Sci..

[cit273] Chang Y., Carroll B. L., Liu J. (2021). Trends Microbiol..

[cit274] Mirvakili S. M., Hunter I. W. (2018). Adv. Mater..

[cit275] Mei T., Zhang H., Xiao K. (2022). ACS Nano.

[cit276] Kim J.-H., Kim J. (2023). Sens. Actuators, B.

[cit277] He K., Liu Y., Wang M., Chen G., Jiang Y., Yu J., Wan C., Qi D., Xiao M., Leow W. R., Yang H., Antonietti M., Chen X. (2020). Adv. Mater..

[cit278] Qiao N., Mostafa H., Corradi F., Osswald M., Stefanini F., Sumislawska D., Indiveri G. (2015). Front. Neurosci..

[cit279] Furber S. (2016). J. Neural. Eng..

[cit280] Kim Y., Chortos A., Xu W., Liu Y., Oh J. Y., Son D., Kang J., Foudeh A. M., Zhu C., Lee Y., Niu S., Liu J., Pfattner R., Bao Z., Lee T.-W. (2018). Science.

[cit281] Wang T., Wang M., Wang J., Yang L., Ren X., Song G., Chen S., Yuan Y., Liu R., Pan L., Li Z., Leow W. R., Luo Y., Ji S., Cui Z., He K., Zhang F., Lv F., Tian Y., Cai K., Yang B., Niu J., Zou H., Liu S., Xu G., Fan X., Hu B., Loh X. J., Wang L., Chen X. (2022). Nat. Electron..

[cit282] Liu Y., Wang H., Shi W., Zhang W., Yu J., Chandran B. K., Cui C., Zhu B., Liu Z., Li B., Xu C., Xu Z., Li S., Huang W., Huo F., Chen X. (2016). Angew. Chem., Int. Ed..

